# Genome-wide meta-analysis of quantitatively measured generalized anxiety symptoms in individuals of European ancestry

**DOI:** 10.1038/s41562-026-02476-7

**Published:** 2026-06-09

**Authors:** Megan Skelton, Brittany L. Mitchell, Elham Assary, Danyang Li, Genevieve Morneau-Vaillancourt, Alan E. Murphy, Abigail R. ter Kuile, Rujia Wang, Mark J. Adams, Enda M. Byrne, Elizabeth C. Corfield, Poppy Z. Grimes, Laurie J. Hannigan, Jihua Hu, Kadri Kõiv, Alex S. F. Kwong, Sergi Papiol, Johanne H. Pettersen, Giorgio Pistis, Enrique Castelao, Nora I. Strom, Peter J. van der Most, Anxiety Disorders Working Group of the Psychiatric Genomics Consortium, Brittany L. Mitchell, Rujia Wang, Mark J. Adams, Enda M. Byrne, Elizabeth C. Corfield, Laurie J. Hannigan, Alex S. F. Kwong, Giorgio Pistis, Enrique Castelao, Nora I. Strom, Silviu-Alin Bacanu, Rosa Cheesman, Kirstin L. Purves, Hüseyin Gedik, Annika B. Faucon, Kritika Singh, Lucia Colodro-Conde, Kristi Krebs, Per Hoffmann, Stefan Herms, Jan Gehlen, Stephan Ripke, Swapnil Awasthi, Teemu Palviainen, Elisa M. Tasanko, Roseann E. Peterson, Daniel E. Adkins, Andrey A. Shabalin, Matthew H. Iveson, Archie Campbell, Laurent F. Thomas, Bendik S. Winsvold, Ole Kristian Drange, Sigrid Børte, Abigail R. ter Kuile, Tan-Hoang Nguyen, Sandra M. Meier, Daniel F. Levey, Darina Czamara, Karmel W. Choi, Baptiste Couvy-Duchesne, Sandra Van der Auwera, Alexander Teumer, Robert Karlsson, Miguel Garcia-Argibay, Donghyung Lee, Ottar Bjerkeset, Eystein Stordal, Julia Bäckmann, Giovanni A. Salum, Clement C. Zai, James L. Kennedy, Gwyneth Zai, Arun K. Tiwari, Stefanie Heilmann-Heimbach, Börge Schmidt, Jaakko Kaprio, Martin M. Kennedy, Joseph Boden, Christel M. Middeldorp, Fabiana L. Lopes, Nirmala Akula, Francis J. McMahon, Elisabeth B. Binder, Lydia Fehm, Andreas Ströhle, Henning Tiemeier, Dan J. Stein, David Whiteman, Catherine Olsen, Zachary Fuller, Xin Wang, Glyn Lewis, Nicholas J. Timpson, Lea K. Davis, Nathan A. Gillespie, Lili Milani, Johannes Schumacher, David P. Woldbye, Andreas J. Forstner, Markus M. Nöthen, Iiris Hovatta, John Horwood, Hermine H. Maes, John-Anker Zwart, Ole Mors, Anders D. Børglum, Preben B. Mortensen, Helga Ask, Ted Reichborn-Kjennerud, Jackob M. Najman, Murray B. Stein, Joel Gelernter, Yuri Milaneschi, Brenda W. Penninx, Dorret I. Boomsma, Eduard Maron, Christian Rück, Tilo T. Kircher, Christiane A. Melzig, Georg W. Alpers, Volker Arolt, Jordan W. Smoller, Annemarie I. Luik, Hans J. Grabe, Henrik Larsson, Patrik K. Magnusson, Anna R. Docherty, Hilary Coon, Rupert Conrad, Manuel Mattheisen, Ole A. Andreassen, Angelika Erhardt-Lehmann, Alexandra Havdahl, Brad Verhulst, Heike Weber, William E. Copeland, Jürgen Deckert, Katharina Domschke, Ian B. Hickie, Kelli Lehto, Michelle K. Lupton, Sarah E. Medland, Andrew M. McIntosh, Albertine J. Oldehinkel, Martin Preisig, Andreas Reif, Naomi R. Wray, Catharina A. Hartman, Nicholas G. Martin, John M. Hettema, Gerome Breen, Thalia C. Eley, GLAD+ authors, Megan Skelton, Danyang Li, Rujia Wang, Kirstin L. Purves, Abigail R. ter Kuile, Matthew Hotopf, Ian R. Jones, Daniel J. Smith, Allan H. Young, Antony J. Cleare, Katrina A. S. Davis, Georgina Krebs, Katharine A. Rimes, David Veale, Roland Zahn, Molly R. Davies, Gursharan Kalsi, Shannon Bristow, Susannah C. B. Curzons, Henry C. Rogers, Katherine N. Thompson, Brett N. Adey, Ian Marsh, Dina Monssen, Monika McAtarsney-Kovacs, Charles J. Curtis, Jahnavi Arora, Saakshi Kakar, Laura Meldrum, Iona Smith, Le Roy Dowey, Victor Gault, Donald M. Lyall, Ruth K. Price, Keith G. Thomas, Zain Ahmad, Helena L. Davies, Christopher Hübel, Sang Hyuck Lee, Yuhao “Leo” Lin, Jared G. Maina, Jessica Mundy, Alish Palmos, Alicia J. Peel, Christopher Rayner, Johan Zvrskovec, Chérie Armour, Andrew M. McIntosh, James T. R. Walters, Gerome Breen, Jonathan R. I. Coleman, Thalia C. Eley, Lifelines Cohort Study, Raul Aguirre-Gamboa, Patrick Deelen, Lude Franke, Jan A. Kuivenhoven, Esteban A. Lopera Maya, Ilja M. Nolte, Serena Sanna, Morris A. Swertz, Peter M. Visscher, Judith M. Vonk, Cisca Wijmenga, Harold Snieder, Naomi R. Wray, NIHR BioResource, John Bradley, Nathalie Kingston, Hannah Stark, Carola Kanz, Alexei Moulton, Nigel Ovington, Jacinta Lee, Debbie Clapham-Riley, Katie Mills, PROTECT-AD Consortium, Andreas Ströhle, Tilo T. Kircher, Uli Wittchen, Andre Pittig, Ingmar Heinig, Jürgen Hoyer, Thomas Fydrich, Alfons Hamm, Jan Richter, Volker Arolt, Katja Kölkebeck, Sylvia Schneider, Jürgen Margraf, Benjamin Straube, Winfried Rief, Paul Pauli, Peter Neudeck, Udo Dannlowski, Jürgen Deckert, Katharina Domschke, Ulrike Lueken, Ole A. Andreassen, Angelika Erhardt-Lehmann, Alexandra Havdahl, Nathan Skene, Brad Verhulst, Heike Weber, Chérie Armour, Helga Ask, William E. Copeland, Udo Dannlowski, Jürgen Deckert, Katharina Domschke, Ian B. Hickie, Kelli Lehto, Tina B. Lonsdorf, Ulrike Lueken, Michelle K. Lupton, Sarah E. Medland, Andrew M. McIntosh, Albertine J. Oldehinkel, Martin Preisig, Andreas Reif, Harold Snieder, James T. R. Walters, Naomi R. Wray, Catharina A. Hartman, Nicholas G. Martin, John M. Hettema, Gerome Breen, Jonathan R. I. Coleman, Thalia C. Eley

**Affiliations:** 1 Social, Genetic and Developmental Psychiatry Centre, Institute of Psychiatry, Psychology and Neuroscience, King’s College London, London, UK; 2 Brain and Mental Health Program, QIMR Berghofer Medical Research Institute, Brisbane, Queensland Australia; 3 School of Biomedical Sciences, Faculty of Medicine, The University of Queensland, Brisbane, Queensland Australia; 4 School of Biomedical Sciences, Queensland University of Technology, Brisbane, Queensland Australia; 5 Research Centre of the Montreal University Institute of Mental Health, University of Montreal, Montreal, Quebec Canada; 6 UK Dementia Research Institute at Imperial College London, London, UK; 7 Department of Brain Sciences, Imperial College London, London, UK; 8 Department of Clinical, Educational, and Health Psychology, University College London, London, UK; 9 National Institute for Health and Care Research Maudsley Biomedical Research Centre, South London and Maudsley NHS Trust, London, UK; 10 Division of Psychiatry, Centre for Clinical Brain Sciences, University of Edinburgh, Edinburgh, UK; 11 The University of Queensland, Child Health Research Centre, Brisbane, Queensland Australia; 12 PsychGen Centre for Genetic Epidemiology and Mental Health, Norwegian Institute of Public Health, Oslo, Norway; 13 Psychiatric Genetic Epidemiology Group, Research Department, Lovisenberg Diaconal Hospital, Oslo, Norway; 14 Population Health Sciences, Bristol Medical School, University of Bristol, Bristol, UK; 15 MRC Integrative Epidemiology Unit, Bristol Medical School, University of Bristol, Bristol, UK; 16 Estonian Genome Centre, Institute of Genomics, University of Tartu, Tartu, Estonia; 17 Department Clinical Translation, Max Planck Institute of Psychiatry, Munich, Germany; 18 Instituto de Salud Carlos III, Centro de Investigación Biomédica en Red de Salud Mental (CIBERSAM), Madrid, Spain; 19 Department of Psychiatry, Lausanne University Hospital (CHUV) and University of Lausanne, Prilly, Switzerland; 20 Department of Psychology, Humboldt-Universität zu Berlin, Berlin, Germany; 21 Institute of Psychiatric Phenomics and Genomics, LMU Munich, University Hospital, Munich, Germany; 22 Centre for Psychiatry Research, Department of Clinical Neuroscience, Karolinska Institutet and Stockholm Health Care Services, Solna, Sweden; 23 Department of Biomedicine, Aarhus University, Aarhus, Denmark; 24 Department of Epidemiology, University Medical Center Groningen, University of Groningen, Groningen, the Netherlands; 25 Centre for Precision Psychiatry, Institute of Clinical Medicine, University of Oslo, Oslo, Norway; 26 Division of Mental Health and Addiction, Oslo University Hospital, Oslo, Norway; 27 Department of Psychiatry, Psychosomatics and Psychotherapy, University Hospital of Würzburg, Würzburg, Germany; 28 PROMENTA Research Centre, Department of Psychology, University of Oslo, Oslo, Norway; 29 Department of Psychiatry and Behavioral Sciences, Texas A&M University, Bryan, TX USA; 30 Stress, Trauma and Related Conditions Research Centre (STARC), School of Psychology, Queen’s University Belfast, Belfast, UK; 31 Department of Psychiatry, University of Vermont, Burlington, VT USA; 32 Institute for Translational Psychiatry, University of Münster, Münster, Germany; 33 Department of Psychiatry, Medical School and University Medical Center OWL, Protestant Hospital of the Bethel Foundation, Bielefeld University, Bielefeld, Germany; 34 German Center for Mental Health (DZPG), Site Jena Magdeburg Halle, Jena, Germany; 35 Center for Intervention and Research on Adaptive and Maladaptive Brain Circuits Underlying Mental Health (C-I-R-C), Site Jena Magdeburg Halle, Jena, Germany; 36 Institute of Clinical Epidemiology and Biometrics, University of Würzburg, Würzburg, Germany; 37 Department of Psychiatry and Psychotherapy, Faculty of Medicine, Medical Center – University of Freiburg, University of Freiburg, Freiburg, Germany; 38 German Center for Mental Health (DZPG), Partner Site Berlin-Potsdam, Berlin, Germany; 39 Brain and Mind Centre, The University of Sydney, Sydney, New South Wales Australia; 40 Experimental Medicine, Systems Neuroscience, University Medical Center Hamburg Eppendorf, Hamburg, Germany; 41 Psychology, Biological Psychology and Cognitive Neuroscience, University of Bielefeld, Bielefeld, Germany; 42 School of Psychology, The University of Queensland, Brisbane, Queensland Australia; 43 School of Psychology and Counselling, Queensland University of Technology, Brisbane, Queensland Australia; 44 Department of Psychiatry, Interdisciplinary Center Psychopathology and Emotion Regulation, University Medical Center Groningen, University of Groningen, Groningen, the Netherlands; 45 Department of Psychiatry, Psychosomatic Medicine and Psychotherapy, University Medical Centre Frankfurt, Frankfurt am Main, Germany; 46 Fraunhofer Institute for Translational Medicine and Pharmacology ITMP, Frankfurt am Main, Germany; 47 National Centre for Mental Health and Centre for Neuropsychiatric Genetics and Genomics, Cardiff University, Cardiff, UK; 48 Institute for Molecular Bioscience, University of Queensland, Brisbane, Queensland Australia; 49 Department of Psychiatry, University of Oxford, Oxford, UK; 50 Psychiatry, Virginia Commonwealth University, Richmond, VA USA; 51 Institute for Genomics in Health, Department of Psychiatry and Behavioral Sciences, State University of New York Downstate Health Sciences University, Brooklyn, NY USA; 52 Life Sciences, Integrative Life Sciences Doctoral Program, Virginia Commonwealth University, Richmond, VA USA; 53 Human and Molecular Genetics, Virginia Institute for Psychiatric and Behavioral Genetics, Virginia Commonwealth University, Richmond, VA USA; 54 Division of Medicine, Human Genetics, Vanderbilt University, Nashville, TN USA; 55 Division of Genetic Medicine, Vanderbilt University Medical Center, Nashville, TN USA; 56 Vanderbilt Genetics Institute, Vanderbilt University Medical Center, Nashville, TN USA; 57 Institute of Human Genetics, University of Bonn, School of Medicine and University Hospital Bonn, Bonn, Germany; 58 Department of Biomedicine, Human Genomics Research Group, University of Basel; University Hospital Basel, Basel, Switzerland; 59 Institute of Medical Genetics and Pathology, Medical Faculty, University Hospital Basel, Basel, Switzerland; 60 Center for Human Genetics, University of Marburg, Marburg, Germany; 61 Department of Psychiatry and Psychotherapy, Charité – Universitätsmedizin, Berlin, Germany; 62 Analytic and Translational Genetics Unit, Massachusetts General Hospital, Boston, MA USA; 63 Helsinki Institute of Life Science, Institute for Molecular Medicine Finland – FIMM, University of Helsinki, Helsinki, Finland; 64 Faculty of Medicine, Department of Psychology and Logopedics, SleepWell Research Program, University of Helsinki, Helsinki, Finland; 65 School of Medicine, Department of Psychiatry, University of Utah, Salt Lake City, UT USA; 66 College of Medicine and Veterinary Medicine, Institute of Genetics and Cancer, University of Edinburgh, Edinburgh, UK; 67 Department of Clinical and Molecular Medicine, Norwegian University of Science and Technology, Trondheim, Norway; 68 HUNT Center for Molecular and Clinical Epidemiology, Department of Public Health and Nursing, Faculty of Medicine and Health Sciences, Norwegian University of Science and Technology, Trondheim, Norway; 69 BioCore – Bioinformatics Core Facility, Norwegian University of Science and Technology, Trondheim, Norway; 70 Clinic of Laboratory Medicine, St. Olavs Hospital, Trondheim University Hospital, Trondheim, Norway; 71 Division of Clinical Neuroscience, Department of Research and Innovation, Oslo University Hospital, Oslo, Norway; 72 Department of Public Health and Nursing, HUNT Center for Molecular and Clinical Epidemiology, Norwegian University of Science and Technology, Trondheim, Norway; 73 Department of Neurology, Oslo University Hospital, Oslo, Norway; 74 Department of Mental Health, Norwegian University of Science and Technology, Trondheim, Norway; 75 Division of Mental Health, St. Olavs Hospital, Trondheim University Hospital, Trondheim, Norway; 76 NORMENT Centre, University of Oslo, Oslo, Norway; 77 Department of Psychiatry, Sørlandet Hospital, Kristiansand, Norway; 78 Division of Clinical Neuroscience, Department of Research and Innovation, Musculoskeletal Health, Oslo University Hospital, Oslo, Norway; 79 Faculty of Medicine, Institute of Clinical Medicine, University of Oslo, Oslo, Norway; 80 Psychiatry, Dalhousie University, Halifax, Nova Scotia Canada; 81 Department of Psychiatry, Division of Human Genetics, Yale University School of Medicine, New Haven, CT USA; 82 Psychiatry, Research, Veterans Affairs Connecticut Healthcare System, West Haven, CT USA; 83 Department of Genes and Environment, Max Planck Institute of Psychiatry, Munich, Germany; 84 Psychiatry, Center for Precision Psychiatry, Massachusetts General Hospital, Boston, MA USA; 85 Psychiatry, Psychiatric and Neurodevelopmental Genetics Unit, Center for Genomic Medicine, Massachusetts General Hospital, Boston, MA USA; 86 ARAMIS laboratory, Paris Brain Institute, Paris, France; 87 Department of Psychiatry and Psychotherapy, University Medicine Greifswald, Greifswald, Germany; 88 Institute for Community Medicine, University Medicine Greifswald, Greifswald, Germany; 89 Department of Medical Epidemiology and Biostatistics, Karolinska Institutet, Stockholm, Sweden; 90 School of Medical Sciences, Faculty of Medicine and Health, Örebro University, Örebro, Sweden; 91 Department of Statistics, Miami University, Oxford, OH USA; 92 Faculty of Nursing and Health Science, Nord University, Levanger, Norway; 93 Department of Psychiatry, Hospital Namsos, Nord-Trøndelag Health Trust, Namsos, Norway; 94 Department of Psychiatry, Universidade Federal do Rio Grande do Sul, Porto Alegre, Brazil; 95 Child Psychiatry, National Institute of Developmental Psychiatry, São Paulo, Brazil; 96 Tanenbaum Centre for Pharmacogenetics, Molecular Brain Sciences Department, Campbell Family Mental Health Institute, Centre for Addiction and Mental Health, Toronto, Ontario Canada; 97 Department of Psychiatry, Division of Neurosciences and Clinical Translation, University of Toronto, Toronto, Ontario Canada; 98 Institute of Medical Science, University of Toronto, Toronto, Ontario Canada; 99 Laboratory Medicine and Pathobiology, University of Toronto, Toronto, Ontario Canada; 100 Stanley Center for Psychiatric Research, Broad Institute of Harvard and MIT, Cambridge, MA USA; 101 Institute for Medical Informatics, Biometry and Epidemiology, University Hospital of Essen, University of Duisburg-Essen, Essen, Germany; 102 Pathology and Biomedical Science, University of Otago, Christchurch, New Zealand; 103 Psychological Medicine, University of Otago, Christchurch, New Zealand; 104 Child and Youth Mental Health Service, Children’s Health Queensland Hospital and Health Service, Brisbane, Queensland Australia; 105 National Institute of Mental Health, Human Genetics Branch, National Institutes of Health, Bethesda, MD USA; 106 Department of Psychiatry and Human Behavior, Alpert Medical School of Brown University, Providence, RI USA; 107 National Institute of Mental Health, Genetic Basis of Mood and Anxiety Disorders, National Institutes of Health, Bethesda, Maryland USA; 108 Psychiatry and Behavioral Sciences, Johns Hopkins University, Baltimore, MD USA; 109 Department of Psychology, Zentrum für Psychotherapie, Humboldt-Universität zu Berlin, Berlin, Germany; 110 Social and Behavioral Science, T.H. Chan School of Public Health, Harvard University, Boston, MA USA; 111 Child and Adolescent Psychiatry, Erasmus University Medical Center, Rotterdam, Netherlands; 112 SAMRC Unit on Risk and Resilience in Mental Disorders, Department of Psychiatry and Neuroscience Institute, University of Cape Town, Cape Town, South Africa; 113 Population Health Program, QIMR Berghofer Medical Research Institute, Brisbane, Queensland Australia; 114 23andMe, Sunnyvale, CA USA; 115 UCL Division of Psychiatry, University College London, London, UK; 116 Department of Neuroscience, Laboratory of Neural Plasticity, University of Copenhagen, Copenhagen, Denmark; 117 Institute of Neuroscience and Medicine (INM-1), Research Center Jülich, Jülich, Germany; 118 Faculty of Medicine, Department of Psychology and Logopedics and SleepWell Research Program, University of Helsinki, Helsinki, Finland; 119 Massey Cancer Center, Virginia Commonwealth University, Richmond, VA USA; 120 Department of Psychiatry, Psychosis Research Unit, Aarhus University Hospital, Aarhus, Denmark; 121 The Lundbeck Foundation Initiative for Integrative Psychiatric Research, iPSYCH, Aarhus University, Aarhus, Denmark; 122 Center for Genomics and Personalised Medicine, Aarhus University, Aarhus, Denmark; 123 The National Centre for Register-based Research, Aarhus University, Aarhus, Denmark; 124 Faculty of Medicine, School of Public Health, University of Queensland, Herston, Queensland Australia; 125 Psychiatry, University of California San Diego, La Jolla, CA USA; 126 School of Public Health, University of California San Diego, La Jolla, CA USA; 127 Psychiatry Research, Veterans Affairs Connecticut Healthcare System, West Haven, CT USA; 128 Departments of Genetics and Neuroscience, Yale University of Medicine, New Haven, CT USA; 129 Amsterdam Neuroscience, Amsterdam Public Health, Amsterdam University Medical Center, Amsterdam, Netherlands; 130 Twin Register and Department of Complex Trait Genetics, Center for Neurogenomics and Cognitive Research, Vrije Universiteit Amsterdam, Amsterdam, the Netherlands; 131 Amsterdam Public Health, Amsterdam University Medical Center, Amsterdam, the Netherlands; 132 Psychiatry, University of Tartu, Tartu, Estonia; 133 Department of Medicine, Centre for Neuropsychopharmacology, Division of Brain Sciences, Imperial College London, London, UK; 134 Department of Psychiatry and Psychotherapy, University of Marburg–UKGM, Marburg, Germany; 135 Department of Psychology, Clinical Psychology, University of Marburg, Marburg, Germany; 136 Department of Psychology, Biological and Clinical Psychology, University of Greifswald, Greifswald, Germany; 137 School of Social Sciences, Department of Psychology, University of Mannheim, Mannheim, Germany; 138 Department of Mental Health, Institute for Translational Psychiatry, University of Münster, Münster, Germany; 139 Epidemiology, Erasmus University Medical Center, Rotterdam, Netherlands; 140 School of Medicine, Psychiatry, University of Utah, Salt Lake City, UT USA; 141 School of Medicine, Psychiatry, Huntsman Mental Health Institute, University of Utah, Salt Lake City, UT USA; 142 Department of Psychosomatic Medicine and Psychotherapy, University Hospital Münster, Münster, Germany; 143 Community Health and Epidemiology, Dalhousie University, Halifax, Nova Scotia Canada; 144 Computer Science, Dalhousie University, Halifax, Nova Scotia Canada; 145 Institute of Psychiatry, Psychology and Neuroscience, King’s College London, London, UK; 146 Division of Psychological Medicine and Clinical Neurosciences, Cardiff University, Cardiff, UK; 147 School of Biomedical Sciences, Ulster University, Coleraine Campus, Northern Ireland, UK; 148 GreenLight Pharmaceuticals Limited, Dublin, Ireland; 149 School of Health and Wellbeing, University of Glasgow, Glasgow, UK; 150 University Medical Center Groningen, University of Groningen, Groningen, the Netherlands; 151 Big Data Institute, Li Ka Shing Centre for Health Information and Discovery, Nuffield Department of Population Health, University of Oxford, Oxford, UK; 152 NIHR BioResource, Cambridge University Hospitals, Cambridge Biomedical Campus, Cambridge, UK; 153 Psychiatric University Hospital, Ludwig-Maximilians-University München, Munich, Germany; 154 Translational Psychotherapy, Institute of Psychology, University of Göttingen, Göttingen, Germany; 155 Institute for Clinical Psychology and Psychotherapy, Technical University of Dresden, Dresden, Germany; 156 Department of Experimental Psychopathology, University of Hildesheim, Hildesheim, Germany; 157 Protestant Hospital of the Bethel Foundation, Department of Psychiatry and Psychotherapy, Bielefeld University, Medical School and University Medical Center OWL, Bielefeld, Germany; 158 Mental Health Research and Treatment Center, Department of Clinical Psychology and Psychotherapy, Ruhr-University Bochum, Bochum, Germany; 159 Department of Psychology I (Biological Psychology, Clinical Psychology and Psychotherapy), Institute of Psychology, University of Würzburg, Würzburg, Germany; 160 Protect-AD Study Site Cologne, Cologne, Germany

**Keywords:** Anxiety, Quantitative trait

## Abstract

Anxiety is heritable and exists on a continuum, with symptoms ranging from adaptive threat response to clinical disorder. Here we performed a genome-wide association meta-analysis of generalized anxiety symptom severity in 693,869 individuals of European ancestry from 14 cohorts. We identified 80 independent genome-wide significant variants within 74 loci, 39 of which were newly associated with anxiety. SNP-based heritability was 5.9% (posterior s.d. = 0.15%). Polygenic scores were significantly associated with anxiety symptom severity and disorder in European, African and South Asian ancestry samples (*R*^2^ = 1.2–2.9%). Significant genetic correlations (*r*_g_) were estimated with mental and physical health traits, including case–control anxiety, neuroticism and depression (*r*_g_ = 0.71–0.85), irritable bowel syndrome (*r*_g_ = 0.57), coronary artery disease, endometriosis and migraine (*r*_g_ = 0.20–0.27). Gene-based and pathway analyses implicated synaptic and axonal processes, with enriched expression in the brain. These findings highlight the discovery power gained from analysing a quantitative trait rather than a case–control phenotype in anxiety genetics.

## Abstract (web summary)

Analysing generalized anxiety symptoms as a quantitative trait rather than a clinical diagnosis, Skelton, Mitchell and colleagues report a genome-wide meta-analysis of 693,869 individuals identifying 74 associated genetic loci, with 39 loci newly associated with anxiety.

## Main

Anxiety disorders are the most prevalent mental health conditions worldwide^1^ and rates are rising^2–4^. Anxiety is associated with reduced quality of life^5^, elevated mortality^6,7^ and is frequently comorbid with other mental^8–12^ and physical^13^ health conditions, ranging from irritable bowel syndrome to cancer. When co-occurring with other conditions, anxiety symptoms can exert an independent and sometimes greater impact on quality of life and functioning than the primary diagnosis^8^, as has been reported in autism^14^ and bipolar disorder^15^.

Twin and family studies estimate the heritability of anxiety disorders at 20–60% (ref. ^13^), with measures capturing stable anxiety typically showing higher heritability^16^. Early case–control genome-wide association studies (GWAS)^17–19^, which aggregated across anxiety subtypes, identified a handful of risk loci. More recent large-scale efforts using a range of analytical approaches have reported between 14 and 51 associated loci^20–22^. A recent GWAS^23^ of anxiety disorders from the Anxiety Disorders Working Group of the Psychiatric Genomics Consortium (PGC-ANX) identified 58 independent loci from over 120,000 cases. Across these studies, single nucleotide polymorphism (SNP)-based heritability estimates ranged from 5% to 10% (refs. ^20,22,23^).

Fear and worry serve an evolutionary function by promoting vigilance and caution in response to potential threats^24^. Variation in threat sensitivity across individuals may be adaptive at the group level and, consistent with this, anxiety symptoms exist in the population along a continuum of frequency and severity. Clinical anxiety represents a practical threshold at the upper extreme of this distribution based on levels of distress and functional impairment. GWAS of anxiety using quantitative symptom scores capture genetic variation across the full phenotypic range, not only at clinical thresholds. This approach can offer greater statistical power^25^ and a more comprehensive representation of genetic associations with anxiety traits. The degree of genetic overlap between quantitative anxiety symptom severity and disorder status has received little study. A GWAS of a 2-item anxiety scale in the European ancestry subsample of the Million Veteran Program (MVP) identified 5 significant loci^26^ and moderate-to-strong genetic correlations with lifetime anxiety disorder (*r*_g_ = 0.59–0.87)^13,26^. However, the brevity of the measure may have limited the potential to comprehensively capture phenotypic variance. Additional support for shared genetic signal across the anxiety continuum, including above and below diagnostically relevant thresholds, comes from a UK Biobank study^19^ reporting high genetic correlations between mild, moderate and severe anxiety symptom groupings (*r*_g_ = 0.76–0.98). Among individuals with lifetime anxiety or depression, anxiety symptom severity has also shown a significant genetic correlation with functional impairment ratings (*r*_g_ = 0.79)^27^, which have clinical relevance.

These findings provide preliminary evidence that a well-powered GWAS of quantitative anxiety would identify variants relevant to both symptom severity and clinically defined anxiety. Aside from the MVP analysis of a two-item scale, previous quantitative anxiety GWAS^28,29^ have incorporated other psychiatric or personality traits to maximize statistical power, resulting in findings relating to a broader construct than anxiety symptoms specifically. To address this, we performed a genome-wide association meta-analysis of generalized anxiety disorder (GAD) symptom severity in 693,869 individuals of inferred European ancestry across 14 cohorts from PGC-ANX, building on our previous study^23^ of individuals with anxiety disorder. Most cohorts used the GAD 7-item scale (GAD-7) or conceptually similar self-report measures of recent GAD symptoms. In addition to identifying associated loci, we performed variant- and gene-level investigations, estimated genetic correlations with relevant traits and evaluated polygenic score prediction in independent samples of European, African and South Asian ancestry.

## Results

### Genome-wide association meta-analysis

**Fig. 1:**
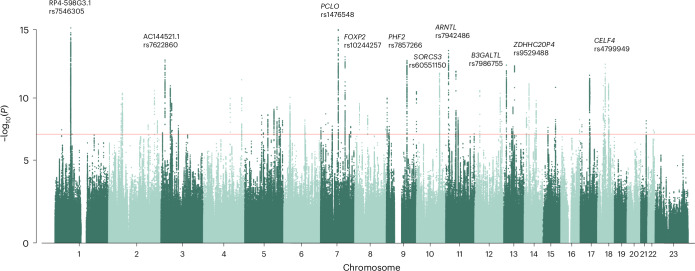
Manhattan plot of the genome-wide association meta-analysis of generalized anxiety symptom severity. Variants are represented as points plotted against their respective genomic location and association significance value (two-tailed test). The red line indicates the genome-wide significant threshold (*P* < 5 × 10^−8^). Sample size, *N* = 693,869.

We performed a genome-wide association meta-analysis of GAD symptom severity in 693,869 individuals of European ancestry across 14 cohorts from 8 countries (see Supplementary Tables 1–3 for cohort, phenotype and GWAS details). The analysis identified 80 independent genome-wide significant variants (*P* < 5 × 10^−8^) across 74 loci (Fig. 1, Supplementary Fig. 1 and Supplementary Table 4. Effect sizes (*β*) represent the associated change in standard deviation units of generalized anxiety symptom score per additional copy of the effect allele. The top signal came from an intergenic locus near the long non-coding RNA *RP4-598G3.1* on chromosome 1 (lead SNP rs7546305; *P* = 3.8 × 10^−15^; *β* = −0.014; 95% confidence interval (CI), −0.017 to −0.010), followed by a locus within an intron of *PCLO* on chromosome 7 (lead SNP rs1476548; *P* = 4.9 × 10^−15^; *β* = −0.014; 95% CI, −0.017 to −0.010). These loci have both previously been associated with internalizing traits, including anxiety (Supplementary Table 5).

Of the 74 loci, 16 had no previous associations with internalizing trait GWAS, including neuroticism, anxiety or depression, at any variant in linkage disequilibrium (LD; defined as *r*^2^ > 0.1) with the lead SNP (Methods and Supplementary Table 5a). Thirty-nine loci were novel for anxiety specifically, measured either as a diagnosis or symptom severity phenotype. Of the 58 loci previously identified in the PGC-ANX anxiety disorder study^23^, all showed the same direction of association in the present analysis, with 19 (33%) reaching genome-wide significance and a further 33 (57%) showing nominal significance (*P* < 0.05) (Supplementary Table 5b). Nine of the 14 cohorts included here also contributed to the PGC-ANX anxiety disorders study, although cohort sample composition differed somewhat due to the availability of symptom score versus diagnostic information. Power calculations^30^ confirmed increased power in the present analysis relative to the anxiety disorder study (Supplementary Table 6).

Among the 6,012 genome-wide significant SNPs, between-study heterogeneity accounted for a moderate proportion of variance in effect size estimates (measured with *I*^2^), with a median *I*^2^ across cohorts of 29.7%. In contrast, the median *I*^2^ for the 80 LD-independent genome-wide significant SNPs was 0%, indicating highly consistent effect size estimates across cohorts. Heterogeneity statistics for the lead SNPs are presented in Supplementary Table 4. Genetic correlations between sufficiently powered cohorts, estimated using LD score regression (LDSC), ranged from 0.64 to 0.97 (significant at a Bonferroni-corrected threshold *P* < 4.6 × 10^−4^; Supplementary Table 7). As a sensitivity analysis, we categorized cohorts by generalized anxiety symptom severity measure (for example, GAD-7; six subgroups) and by ascertainment method (community and clinical subgroups) (Supplementary Table 2). Subgroup meta-analyses were performed as per the main analysis, and genetic correlations between subgroups were estimated with LDSC. While most subgroup comparisons were underpowered, including by ascertainment method, the genetic correlation between the 2 most frequently used measures was high (GAD-7 and GAD-2, *r*_g_ = 0.86; 95% CI, 0.79–0.92; *P* = 9.6 × 10^−166^, significant at a Bonferroni-corrected threshold *P* < 5 × 10^−3^; Supplementary Table 8).

### SNP-based heritability and genetic correlations with external traits

**Fig. 2:**
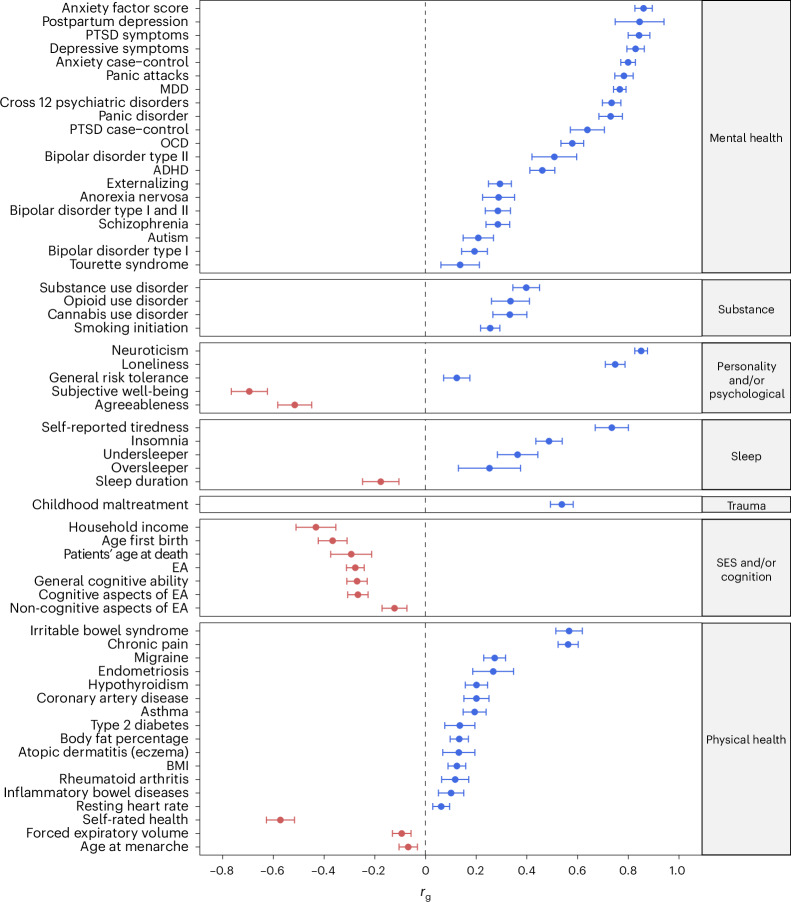
Genetic correlations between generalized anxiety symptom severity meta-analysis and a range of traits from existing GWASs. Genetic correlations estimated using LDSC (*N* = 693,869). All correlations shown are significant at a Bonferroni-corrected threshold for 105 tests (2-tailed *P* < 4.76 × 10^−4^). Points represent genetic correlation estimates and bars represent 95% CIs. Exact *P* values and full results for all tested traits are provided in Supplementary Table 9. Forced expiratory volume is a measure of lung function. ADHD, attention-deficit/hyperactivity disorder; BMI, adult body mass index; EA, educational attainment; MDD, major depressive disorder; OCD, obsessive–compulsive disorder; PTSD, post-traumatic stress disorder; SES, socio-economic status.

The SNP-based heritability estimate from SBayesRC was 5.90% (posterior s.d. = 0.146%). SBayesRC was selected due to the known underestimation of heritability from LDSC^31^. LDSC^32^ indicated that genomic inflation was largely not attributable to confounding (intercept = 1.03; 95% CI, 1.01–1.05). To characterize the broader genetic architecture of GAD symptoms, we used LDSC^33^ to estimate genetic correlations with 105 traits spanning mental and physical health, personality, cognitive and socio-economic domains (Supplementary Table 9). Following Bonferroni correction (*P* < 4.8 × 10^−4^), 64 associations remained significant (Fig. 2, omitting 4 traits represented by multiple studies).

The strongest correlations were observed with quantitative internalizing traits, including neuroticism, depressive symptoms and a genetic anxiety factor (*r*_g _= 0.83–0.86), and with case–control phenotypes for anxiety and depression (*r*_g _= 0.71–0.80). Moderate-to-strong estimates were also found for post-traumatic stress disorder (*r*_g_ = 0.64), insomnia (*r*_g_ = 0.49), irritable bowel syndrome (*r*_g_ = 0.57) and chronic pain (*r*_g_ = 0.56). Negative correlations were observed with socio-economic status indicators including household income (*r*_g_ = −0.43). Smaller but significant correlations were identified with coronary artery disease, endometriosis, hypothyroidism and migraine (*r*_g_ = 0.20–0.27). Most other associations with physical traits and illnesses were comparatively weaker and included positive correlations with resting heart rate, rheumatoid arthritis and atopic dermatitis, and a negative correlation with lung function (absolute *r*_g_ = 0.06–0.13).

### Conditional analyses

To assess whether genome-wide significant loci for generalized anxiety symptoms represented associations unique to this phenotype, we performed multi-trait conditional and joint analysis (mtCOJO)^34^, conditioning on highly genetically correlated traits. mtCOJO uses Generalized Summary-data-based Mendelian Randomization (GSMR) to estimate the effect of a conditioning trait on an outcome (*b*_xy_). Conditioning on neuroticism (67 instruments, GSMR *b*_xy_ = 0.47 ± 0.02) resulted in 9 remaining genome-wide significant SNPs across 2 loci (*P* < 5 × 10^−8^; Supplementary Table 10). Conditioning on case–control anxiety (11 instruments, *b*_xy_ = 0.22 ± 0.02) yielded 66 significant SNPs across 6 loci, and conditioning on case–control depression (98 instruments, *b*_xy_ = 0.31 ± 0.01) produced 76 SNPs across 6 loci. Approximately half of SNP effect sizes were attenuated and standard errors had a median inflation of 7–9%, suggesting the loss of significance was not solely attributable to reduced power. Sign changes were observed in 17–20% of variants for each trait, consistent with instability, as expected under high genetic correlations. The LDSC SNP-based heritability (*h*^2^) estimates of the conditioned summary statistics were significant (neuroticism *h*^2^ = 0.014; 95% CI, 0.012–0.016; *P* = 1.02 × 10^−42^; case–control anxiety *h*^2^ = 0.018; 95% CI, 0.016–0.020; *P* = 1.54 × 10^−59^; case–control depression *h*^2^ = 0.017; 95% CI, 0.015–0.019; *P* = 7.34 × 10^−51^), but block jack-knifing confirmed that each was significantly lower than the original estimate (*Z*-statistic = 33.24, *P* = 3.3 × 10^−242^; *Z* = 28.61, *P* = 5.0 × 10^−180^; *Z* = 30.48, *P* = 4.24 × 10^−204^, respectively).

### Polygenic risk scores

To evaluate the within- and cross-ancestry generalizability of our GWAS findings, we generated a polygenic risk score (PRS) for GAD symptom severity using SBayesRC^35^ and tested its association with both quantitative and case–control anxiety outcomes in independent samples across ancestry groups (Supplementary Table 11). A Bonferroni-corrected significance threshold of *P* < 1.7 × 10^−2^ was applied, accounting for 3 ancestry groups tested per outcome. The PRS significantly explained 2.9% of the variance (*R*^2^) in generalized anxiety symptom scores in an independent European ancestry sample (*β* = 0.55; 95% CI, 0.44–0.65; *P* = 8.9 × 10^−24^), 1.4% in an African ancestry sample (*β* = 0.48; 95% CI, 0.28–0.67; *P* = 2.9 × 10^−6^) and 1.2% in a South Asian ancestry sample (*β* = 0.45; 95% CI, 0.27–0.64; *P* = 2.0 × 10^−6^). For case–control anxiety, assuming 20% prevalence, the PRS explained 1.8% (*β* = 0.25; 95% CI, 0.14–0.36; *P* = 1.2 × 10^−5^) of the variance on the liability scale in a European ancestry sample, 1.7% (*β* = 0.27; 95% CI, 0.10–0.44; *P* = 2.0 × 10^−3^) in an African ancestry sample and 1.4% (*β* = 0.25; 95% CI, 0.10–0.41; *P* = 1.3 × 10^−3^) in a South Asian ancestry sample. These *R*^2^ values represent the variance explained by the PRS beyond that accounted for by covariates.

### Positional and functional annotation

Functionally informed fine-mapping using PolyFun^36^ and SuSiE^37^ identified 4 putative causal variants with posterior inclusion probabilities (PIP) ≥0.95. Two of these were index variants for genome-wide significant loci: rs2392289 at locus 31 (PIP = 0.983) and rs72676302 at locus 58 (PIP = 0.978). The remaining two variants were in loci that did not reach genome-wide significance. One variant, rs72676302, had a high Combined Annotation Dependent Depletion (CADD) score of 19.78, suggesting a deleterious effect (above the suggested threshold of 12.37 (ref. ^38^)), although there was little biological evidence that it is within a regulatory element (regulomeDB score = 5). In addition, we identified 24 small credible causal sets (<10 variants each) that cumulatively met the PIP threshold (Supplementary Table 12).

SNP-level gene annotation was performed using FUMA^39^ based on positional, expression quantitative trait loci (eQTL), and chromatin interaction (Hi-C) mapping (Supplementary Table 13). We identified genes annotated by more than one method, with this convergence providing greater support for their involvement. This approach again highlighted *PCLO* and *CRHR1*, a key regulator of the stress response, along with *TMEM106B*, which has been repeatedly associated with anxiety^18,19,23^ and with depression^40^. *SORCS3* was implicated by both positional and chromatin interaction mapping and has also been reported in a previous anxiety GWAS^23^. In addition, multiple genes were identified that have previously been implicated in depression (for example, *ERBB4*, *GRM7*, *VRK2*, *DCC*, *LRFN5*, *PCLO* and *NEGR1*) and schizophrenia (for example, *ERBB4*, *VRK2* and *NEGR1*). *MAD1L1* (refs. ^17–19,26^) was supported only by positional mapping in our analysis but has been linked to anxiety phenotypes in previous GWAS, lending further support to its potential relevance. Although detailed functional information was limited for many mapped genes, several are posited to play roles in key neurotransmitter systems, including glutamatergic (*GRM7* and *HOMER1*), GABAergic (*ERBB4*) and dopaminergic (*DRD1*) signalling. In contrast, gene annotations for loci novel for internalizing traits did not map clearly to defined neurobiological pathways relevant to anxiety. For example, rs58179213 is proximal to multiple genes implicated in diverse biological processes, including lung function (*SFTPC*), intestinal barrier function and immunoprotective inflammation (*FHIP2B*), and hair growth (*HR*). While such processes could influence anxiety via indirect pathways (for example, gut–brain interactions or somatic symptoms), independent replication will be required to provide the greater power necessary to determine their robustness and relevance.

### Gene-based associations and enrichment

Gene-based association analysis was conducted using MAGMA^41^, which aggregates trait–SNP associations across all SNPs within a gene while accounting for LD. In total, 197 genes across 80 independent loci surpassed the Bonferroni-corrected significance threshold (*P* < 2.5 ×10^−6^; Supplementary Table 14). The top associated gene was *PCLO* (*P* = 2.5 × 10^−21^), mirroring the SNP-based results.

To assess biological pathways significantly enriched for associations with GAD symptom severity, we performed pathway analysis in MAGMA with predefined gene sets (MsigDB^42^; curated and Gene Ontology terms; Supplementary Table 15,a). Six gene sets passed Bonferroni correction (*P* < 2.9 × 10^−6^): postsynaptic membrane (271 genes, *P* = 2.6 × 10^−8^), synaptic membrane (383 genes, *P* = 7.1 × 10^−8^), axon (627 genes, *P* = 5.3 × 10^−7^), neurogenesis (1,627 genes, *P* = 8.6 × 10^−7^), postsynapse (648 genes, *P* = 9.2 × 10^−7^) and generation of neurons (1,412 genes, *P* = 1.0 × 10^−6^). These results were unaffected by the exclusion of the major histocompatibility complex region, although the number of genes per set slightly decreased (Supplementary Table 15b).

Gene–tissue expression analysis was conducted using catalogues of gene expression levels across different human tissues (Supplementary Fig. 2–5), with a Bonferroni correction applied for the number of tests within each tissue set. Among brain samples from 11 developmental stages (BrainSpan), only the 2 prenatal samples were significant (*P* = 6.3 × 10^−4^ and 2.5 × 10^−3^). In adult tissues (GTEx v.8 (ref. ^43^)), significant enrichment was observed in brain (*P* = 3.0 × 10^−9^) and pituitary (*P* = 4.9 × 10^−5^) tissues. Analysis of more specific tissue types revealed enrichment in 11 brain regions, most strongly in the frontal cortex, cortex, cerebellum, anterior cingulate cortex and nucleus accumbens (all *P* < 9.3 × 10^−4^; Supplementary Table 16).

### Drug targets

To explore therapeutic relevance, we ran DrugTargetor^44^ to identify drug priorities with potential utility for clinical anxiety, based on their relevance for GAD symptom severity. We did not observe any significant enrichment of anxiety associations for the 1,551 drug targets tested (Supplementary Table 17). However, at the drug class level, we identified significant associations for Anatomical Therapeutic Chemical classifications N06 ‘psychoanaleptics’, including N06A antidepressants, N02A ‘opioids’ and N06B ‘psychostimulants’ (Bonferroni-adjusted *P* < 0.05; Supplementary Table 18). With the exception of psychostimulants, these classes include drugs that have been used clinically to treat anxiety^45^. The enrichment for psychostimulants probably reflects shared dopaminergic and noradrenergic pathways involved in arousal and vigilance and underlying both anxiety and attention, consistent with pleiotropic genetic associations rather than direct therapeutic action.

## Discussion

In this genome-wide association meta-analysis of 693,869 individuals from 14 cohorts, we identified 80 genome-wide significant variants across 74 loci associated with quantitative measures of GAD symptom severity. Approximately half of the identified loci replicated associations reported in previous anxiety GWAS^19–23,46^, while the remainder were newly identified associations.

The strongest intragenic association was estimated for rs1476548 within *PCLO*, which was also implicated through positional mapping, eQTL mapping and gene-based association testing. *PCLO* encodes a protein involved in regulating presynaptic structure and neurotransmitter release. This gene has long been of interest in major depressive disorder^47^, with recent evidence also linking it to anxiety disorders^20,23^. Another gene of interest from our analysis was *SORCS3*, which was supported by multiple lines of evidence in the recent PGC-ANX case–control anxiety GWAS^23^. *SORCS3* plays a role in postsynaptic functioning and glutamate receptor regulation, particularly in the hippocampus^48^. It has been linked to memory and learning processes, specifically synaptic depression and fear extinction^49^, and mental health and neurodevelopmental conditions, including major depressive disorder, Tourette syndrome, attention deficit hyperactivity disorder and autism^50,51^.

Our SNP-based heritability estimate (5.9%) aligned with an existing GWAS of GAD symptom severity (5.6%)^26^ while remaining lower than liability scale estimates from case–control anxiety meta-analyses^23^. In contrast to traditional case–control phenotyping, which aims to maximize clinical specificity through diagnostic thresholds, our approach leveraged the full spectrum of symptom variability, increasing power for discovery^52^ and capturing genetic risk relevant to both subclinical and clinical presentations. The key differences between clinical diagnoses and symptom severity measures relate to the presence of distress and impairment and to symptom duration. While subclinical symptoms can still cause distress and impairment, this is not always true for lower levels of anxiety severity, potentially contributing to some diagnosis-specific genetic variance. Similarly, diagnostic tools often assess lifetime occurrence and require symptoms to be present for a minimum period of time (6 months for GAD), whereas symptom severity scales typically capture recent experiences (for example, the past 2 weeks), introducing greater susceptibility to transient fluctuations and measurement noise. Consistent with this, GWAS^53–55^ of depression symptom severity typically yield lower SNP-based heritability estimates than case–control analyses. We aimed to partially address and control for temporal fluctuations and better approximate a stable underlying trait^16^ by incorporating assessments from multiple time points into our analysis, where available. In addition, our meta-analysis combined data from multiple cohorts with differences in phenotype definitions, genotyping arrays, imputation methods, quality control procedures and population structure adjustments, which may have introduced further heterogeneity and reduced the observed SNP-based heritability. By contrast, single-cohort studies with individual-level data are more homogeneous and can implement alternatives to summary-statistics-based methods. Despite our lower heritability estimate, the strong genetic correlation observed between our phenotype and case–control anxiety suggests that GAD symptom severity captures much of the same genetic risk. This finding is consistent with a recent analysis of obsessive–compulsive symptoms^56^. Quantitative, symptom-based approaches may be particularly well suited to genetic studies of anxiety, given the high burden of anxiety symptoms observed across other mental health conditions^8–11^. In this context, efforts to isolate ‘pure’ anxiety cases may be both methodologically challenging and capture an unusual clinical phenotype that is unrepresentative for most individuals with anxiety.

There was a broad range of significant genetic correlations across both mental and physical health conditions, consistent with the frequent co-occurrence with anxiety symptoms and widespread pleiotropy. A strong genetic correlation was observed with neuroticism, a well-established risk factor for anxiety^23^. This probably reflects both genuine shared liability and conceptual or item-level overlap between the measures. Conditional analyses indicated extensive overlap between loci associated with generalized anxiety symptoms and those implicated in neuroticism, case–control anxiety and depression. Many genome-wide significant loci may therefore index a broader neuroticism-related liability, although the presence of anxiety-specific associations cannot be excluded due to the statistical noise introduced by conditioning on highly genetically correlated traits. Many of the genetic correlations aligned with findings from a genomic structural equation modelling analysis of anxiety symptoms and disorder^22^, including strong associations with irritable bowel syndrome and chronic pain, and a moderate association with migraine. These results do not necessarily indicate horizontal pleiotropy but could instead arise from the experience of these conditions eliciting uncertainty and worry that contribute to anxiety.

Polygenic scores derived from our genome-wide association meta-analysis demonstrated within- and cross-ancestry generalizability, significantly explaining 1.2–2.9% of the variance in GAD symptom severity in European, African and South Asian ancestry samples. Across these samples, the PRS also accounted for 1.4–1.8% of the variance in case–control anxiety on the liability scale. While broadly consistent with the 0.5–2.3% range reported in the PGC-ANX case–control analysis^23^, direct comparisons are limited by methodological differences in PRS construction and target sample composition. A key limitation of the present meta-analysis is its restriction to cohorts of European ancestry, due to lack of data for other ancestries at sufficient scale for GWAS analysis. Our PRS findings support a degree of shared genetic architecture with African and South Asian populations. Ancestry-specific modelling across diverse populations remains necessary to identify population-specific risk loci, such as that identified in a previous analysis^26^ of African American participants, and ensure equitable benefits from genetic discoveries. Overall, these findings provide additional evidence that quantitative phenotyping can effectively capture genetic signal relevant to clinical anxiety.

The GAD symptom severity measures and ascertainment methods varied across contributing cohorts, although most assessed symptoms using the GAD-7. While widely adopted across clinical and research contexts, the GAD-7 does not comprehensively assess all GAD symptoms from the Diagnostic and Statistical Manual of Mental Disorders (DSM-5)^57^—omitting sleep, fatigue and concentration problems—and is not designed to capture symptoms of fear-based anxiety (that is, phobias and social anxiety disorder) or panic disorder. This limits the generalizability of our findings across anxiety disorders, particularly in light of evidence for partially distinct phenotypic and genetic contributions to GAD compared with fear-based disorders^58,59^. Expanding future studies to incorporate a broader range of anxiety symptom measures will enable more robust, transdiagnostic translation of these findings. Furthermore, future work could examine the genetic architecture of individual symptom domains, such as cognitive versus physiological symptoms, to better understand the biological specificity of these. Our subgroup analyses based on measure and ascertainment method were largely underpowered to reliably estimate SNP-based heritability or correlations. Although sufficiently powered comparisons indicated high genetic overlap, we cannot be certain that all cohorts captured the same underlying genetic architecture. While population-based cohorts allow assessment of the full range of symptoms, symptom severity measures typically better distinguish variation at the upper end of the distribution. This results in highly skewed symptom severity scores, as most participants report few or no symptoms, whereas individuals in clinical cohorts typically report more symptoms. Combining population-based cohorts with studies selecting on diagnosis introduces some heterogeneity and may limit generalizability to broader populations, but it can help yield a more normally distributed phenotype for GWAS analysis. This combination may have improved statistical power for detecting associations in our study.

Our quantitative GWAS of GAD symptom severity identified more genome-wide significant loci than a slightly larger and mostly overlapping case–control anxiety study (*N* = 852,222; 122,341 cases)^23^, with many loci replicated across the 2 methods. This aligns with expectations under the liability-threshold model when considering common conditions such as anxiety, whereby quantitative traits generally offer greater statistical power than case–control designs of equal sample size^52^. Beyond identifying anxiety-associated loci, our results implicate key neurobiological pathways, including synaptic function and neurotransmission, and notable genes such as *PCLO* and *SORCS3*. These findings demonstrate that a quantitative anxiety symptom-based phenotype can reveal biologically meaningful signals and complements insights from case–control designs. Clinically ascertained samples remain essential for identifying disorder-specific biology and mapping genetic risk to diagnostic presentations; however, obtaining clinical cases at sufficient scale for binary genome-wide analyses is challenging. Although electronic health records offer an efficient option, these are limited to individuals seeking and receiving medical attention. Quantitative, symptom-based approaches within biobanks and population studies therefore offer a promising scalable alternative for advancing the field of anxiety genetics. Moving forwards, the combination of these with deeply phenotyped clinical cohorts will be crucial for translating genetic insights into diagnostic and therapeutic advances. Together, these approaches can elucidate the biological continuum of anxiety, from healthy stress responses to debilitating disorder. Given the high and rising rates of anxiety, especially in young adults, it is more important than ever to improve our ability to identify and understand sources of risk. Despite its public health impact, progress in anxiety genetics lags behind other major mental health conditions. We hope our findings encourage genome-wide investigations leveraging existing but potentially underutilised anxiety severity data in genotyped cohorts, accelerating our progress in understanding the genetic architecture of anxiety.

## Methods

### Participants and measures

We meta-analysed data from 14 international cohorts (*N* = 693,869): 13 PGC-ANX studies with generalized anxiety symptom data and summary statistics from a pre-existing GWAS^26^. Details of each study and sample descriptives are provided in Supplementary Table 1. We performed a meta-analysis with access to individual participant data^60^, such that each PGC-ANX cohort performed genome-wide association analyses specifically for this study and shared summary statistics with the core analytical team. The majority of the sample (70%) had completed the GAD-7 or closely related brief self-report measures assessing recent anxiety symptoms. The remaining 30% used other brief self-report anxiety scales (Supplementary Table 1), each available in at least 3,000 individuals. We analysed total sum scores, with higher scores indicating greater severity of symptoms. If participants were missing data on <25% of measure items, the missing scores were imputed with the participant’s mean score of the other items. Participants with ≥25% missing data were excluded from analysis. Several cohorts had assessed anxiety symptoms on two or more occasions. Longitudinal twin studies have shown that symptom stability is primarily driven by genetic factors^16,61,62^ and that stability extracted from repeated assessments yields higher heritability estimates than single time points^16^. For cohorts with anxiety assessments from 3 or more time points (12% of the sample), a latent factor was created in R with the package lavaan^63^, the predict function and a maximum likelihood estimator. For cohorts with 2 time points (44%), a mean score was calculated. All scores, whether single time point, mean or factor score, were standardized to have a mean of zero and a standard deviation of one. Given the high comorbidity of anxiety and other mental health conditions, no additional exclusions were applied beyond those defined by each study. For two cohorts—Genetic Links to Anxiety and Depression Study (GLAD+) and the UK Biobank—individual-level data were merged before the GWAS. Participants from clinical cohorts had been recruited based on a lifetime history of depression or anxiety, as assessed by self-reported diagnostic questionnaires.

### Meta-analysis

Supplementary Table 3 provides details of the studies that contributed to this meta-analysis, which were: Australian Genetics of Depression Study (AGDS)^64^, Avon Longitudinal Study of Parents and Children (ALSPAC)^65,66^, CoLaus|PsyCoLaus^67^, Estonian Biobank^68^, Generation Scotland^69^, NIHR Bioresource GLAD+^70^, Lifelines^71^, MEGA TRR58^72^, MVP^26^, Norwegian Mother, Father and Child Cohort Study^73^, Providing Tools for Effective Care and Treatment of Anxiety Disorders (PROTECT-AD)^74^, Twins Early Development Study (TEDS)^75^, Tracking Adolescents’ Individual Lives’ Survey (TRAILS)^76^ and UK Biobank^77^. Each cohort performed genotyping using microarray platforms and imputed genotypes using ancestry-matched panels, primarily the Haplotype Reference Consortium^78^. Standard quality control procedures were applied, including filters on sample and variant call rates, sex concordance and excessive heterozygosity (full details in Supplementary Table 3). The one set of pre-existing summary statistics was from an analysis in the MVP, obtained through the database of Genotypes and Phenotypes (dbGaP; phs001672). Most groups adopted a mixed linear model approach and retained related individuals in their GWAS. Where applicable, covariates such as ancestry principal components and genotyping batch were included. We did not include age or sex as covariates, as they are not confounders of genetic effects and may represent effect moderators of interest warranting dedicated follow-up investigation, rather than variables to be adjusted for. All resulting summary statistics were on the GRCh37 genome assembly (build 37, hg19). Before meta-analysis, variant-level quality control was performed across the summary statistics, excluding those with minor allele frequency <1% or imputation accuracy score <0.6. Variants present in fewer than half of the contributing cohorts were excluded, resulting in a total of 7,499,431 autosomal SNPs. X-chromosome data were analysed from 7 cohorts (166,852 variants), with male genotypes coded as diploid (0/2) and sex included as a covariate. The meta-analysis was conducted in METAL (v.2020-05-05)^79^ using an inverse-variance-weighted, standard-error-based approach. The β values from the meta-analysis represent the associated change in standard deviation units of generalized anxiety symptom score per additional copy of the effect allele. All statistical tests conducted in this study were two tailed.

Heterogeneity across cohorts was assessed by inspecting the *P* values from Cochran’s *Q* test as implemented in METAL. In addition, we calculated the median *I*^2^ values (HetISq in METAL) for SNPs reaching genome-wide significance (*P* < 5 × 10^−8^) and for independent lead SNPs. We also estimated genetic correlations between contributing cohorts using LDSC^33^. The inclusion of clinical alongside community-based cohorts offered greater representation across the full range of anxiety symptom severity, increasing statistical power as evidenced in a recent depression GWAS^80^. However, due to the risk of bias or confounding from differences in study design and phenotyping, we performed subgroup meta-analyses stratified by anxiety measure and excluding clinical cohorts (Supplementary Table 2). Meta-analyses of the measure and ascertainment subgroups were performed in METAL, and genetic correlations between the groups estimated using LDSC^33^. For both cohort and subgroup genetic correlations, most pairwise comparisons were not sufficiently powered (that is, heritability *z*-scores <4 for 1 or both sets of summary statistics^81^) to draw conclusions.

To identify LD-independent significant SNPs and loci, clumping was performed in FUMA (v.1.6.5)^39^. The *r*^2^ threshold for independent significant SNPs was 0.1, and was 0.05 for lead SNPs, within a 500-kb window. Genome-wide significance was defined using the conventional threshold (*P* < 5 × 10^−8^).

To identify novel loci in our results, we cross-referenced significant loci with published trait associations from the GWAS Catalog^82^ using LDTrait^83^ (date accessed 2 September 2025), applying an *r*^2^ threshold of >0.1 and a 500-kb window. Novelty was strictly defined as having no previous associations with internalizing traits including anxiety, depression, neuroticism and worry. To supplement this, we compared our results with recent anxiety and depression studies^23,80^ not yet available in the GWAS Catalog. Overlapping significant loci were identified with BEDtools (v.2.31.0)^84^ and LD assessed using a threshold of *r*^2^ > 0.1. The investigation of novel loci also revealed the extent to which our results replicated previous findings. We also determined novel loci specifically for anxiety, whether assessed as symptom severity or a case–control phenotype. Many of the 14 cohorts in our meta-analysis overlap with previous case–control anxiety meta-analyses, with the exception of GLAD+, Lifelines, PROTECT-AD, TEDS and MEGA (approximate *N* = 110,000). In some instances, the cohort sample composition differs due to the availability of quantitative versus diagnostic information.

### SNP-based heritability and genetic correlations with external traits

We estimated SNP-based heritability via SBayesRC (v.0.2)^35^. This provided an estimate of the proportion of variance in quantitative anxiety attributable to variation in the common SNPs present in this meta-analysis. We inspected the LDSC^32^ genomic inflation factor (*λ*_GC_) and intercept to evaluate the contribution of potential confounding relative to polygenicity. Genetic correlations were also computed using LDSC with 105 GWAS summary statistics covering a broad range of phenotypes and applying a Bonferroni-corrected *P* value threshold of 4.76 × 10^−4^.

High genetic correlations, such as those often observed between anxiety, depression and neuroticism, do not necessarily indicate identical biology; even when most loci are shared, traits may involve different biological pathways, tissue enrichments or show individual patterns of relationships with other traits. Identifying unique genetic influences on anxiety is important to better understand its specific aetiology and inform potential treatment pathways. However, conditional analyses, especially in the presence of strong genetic correlations, are statistically challenging and require substantial power, and therefore should be interpreted cautiously. We used the mtCOJO^34^ tool from GCTA (v.1.94.1), which performs conditional analyses between summary statistics to provide marginal effect estimates for the trait of interest. We conditioned our anxiety meta-analysis on depression diagnosis^80^ (98 index SNPs), neuroticism^85^ (67 index SNPs) and anxiety diagnosis^23^ (11 index SNPs). Depression symptoms^86^ was underpowered (two index SNPs) for the analysis. We also estimated the SNP-based heritability of each set of conditioned summary statistics with LDSC and used block jack-knifing to compare these to the unconditioned heritability estimate.

### PRSs

To evaluate the within- and cross-ancestry validity of our GWAS, we calculated GAD symptom severity PRSs in independent samples from the UK Biobank^77^ and Prospective Imaging Study of Ageing (PISA)^87^. We then performed regressions between our PRS and quantitative anxiety, using GAD-7 scores, and case–control anxiety, as defined by a self-reported diagnostic questionnaire or self-report of a diagnosis from a health professional. Specifically, we used SBayesRC^35^ to calculate PRSs in European, African and South Asian ancestry samples, excluding related individuals. SBayesRC is a Bayesian regression method that uses GWAS summary statistics to estimate SNP effect sizes while accounting for LD and polygenic architecture. It extends the SBayesR framework by incorporating functional annotations or prior biological information, improving the detection of probable causal variants and enhancing predictive accuracy for complex traits. We conducted linear regressions to assess the variance explained in GAD symptom severity by the PRS in each sample (European *N* = 3,452; African *N* = 1,581; South Asian *N* = 1,813). For case–control status, we performed logistic regressions to estimate associations in the target samples (European total *n* = 3,107, case *n* = 407; African total *n* = 1,303, case *n* = 218; South Asian total *n* = 1,549, case *n* = 265). We calculated Nagelkerke’s *R*^2^ for our PRS on the liability scale using the observed linear regression *R*^2^ and corresponding formula^88^, assuming a population prevalence of 20%. All regressions included the first ten ancestry-specific principal components and genotyping batch as covariates. The variance explained by the PRS was calculated as the difference in *R*^2^ between a full model, including the PRS and covariates, and a null model with only covariates.

### Positional and functional annotation

We used PolyFun (v.1.0.0)^36^ to estimate per-SNP heritabilities, leveraging a regularized extension of stratified-LDSC applied to the v.2.2UKB baseline-LF model annotations, which captures heritability enrichment related to allele frequency, LD and variant function. These prior causal estimates were then used for fine-mapping in SuSiE (v.0.11.92)^37^, limiting to a maximum of one causal SNP per locus. We extracted annotations at a PIP threshold of ≥0.95 and created credible causal sets containing the minimum set of ranked variants that cumulatively met this threshold. Unlike standard definitions of credible causal sets in SuSiE, we did not require a minimum pairwise *r*^2^ between variants in a set, as the PolyFun + SuSiE pipeline does not incorporate LD estimates.

We performed SNP-level gene annotation using FUMA (v.1.6.5)^39^, integrating three complementary methods: positional mapping (based on physical proximity to genes), eQTL mapping (linking variants to gene expression) and chromatin interaction mapping (using Hi-C data to identify regulatory interactions). eQTL mapping used significant SNP–gene pairs and eQTLs from the brain tissue datasets GTEx v.8 Brain^43^ (13 regions) and BRAINEAC^89^ (10 regions), and averaged expressions across these, applying a false discovery rate (FDR) threshold of *P* < 0.05. Chromatin interaction mapping used Hi-C brain tissue data (dorsolateral prefrontal cortex, hippocampus and left and right ventricles)^90^ and adult and fetal cortex^91^, with an FDR threshold of *P* < 1 × 10^−6^. These methods differ in their underlying biological rationale and may implicate different genes. Genes identified by two or more mapping approaches were therefore highlighted, as convergence across the methods increased our confidence in the potential functional relevance of a gene.

### Gene-based associations and enrichment

Gene-based association, gene-set and gene–tissue expression enrichment analyses were performed in MAGMA (v.1.08)^41^ via FUMA (v.1.6.5)^39^. These analyses aimed to identify genes associated with GAD symptom severity, biological pathways enriched for associated genes and relevant tissues where genes are preferentially expressed, offering insight into the potential biological mechanisms underlying our findings. For gene-based associations, we tested 19,954 genes, applying a Bonferroni-corrected significance threshold of *P* < 2.5 × 10^−6^. SNPs were mapped to genes using a 35-kb upstream and 10-kb downstream window. Gene-set analyses were performed using 6,494 curated gene sets (c2.all) and 10,529 Gene Ontology terms (c5.bp, c5.cc and c5.mf) from the Molecular Signatures Database (MSigDB; v.2023.1.Hs)^42^. Significance was determined by a Bonferroni-corrected threshold of *P* < 2.9 × 10^−6^. For tissue enrichment, we tested relationships between trait-associated genes and gene expression in human tissues, using data from BrainSpan (brain samples from 11 general developmental stages and 29 specified ages) and GTEx v.8 (covering 30 general and 54 specific tissue types).

### Drug targets

We examined whether genes associated with GAD symptom severity were associated with individual drugs and drug classes using the DrugTargetor (v.1.3)^44^ method. DrugTargetor integrates MAGMA gene-level association results with curated drug–gene interaction databases (ChEMBL^92,93^ and DGIdb^94^). We used MAGMA (v.1.10) to prioritize associated genes within windows 35 kb upstream and 10 kb downstream. We restricted our analysis to hypothesized drug action within the nervous system, a maximum of 1,551 unique drugs and 163 drug classes. To assess the enrichment of drug classes, we calculated the area under the enrichment curve (AUC), where 50% indicates random enrichment and 100% optimal enrichment, and AUC significance was assessed using one-sided Wilcoxon–Mann–Whitney tests.

### Ethics

This study analysed pre-existing data from cohort studies. Each contributing study obtained ethical approval from the relevant institutional ethics committee, and participants provided informed consent permitting genetic and health-related research. Details of all ethical approvals are provided in Supplementary Table 1, and data application numbers where applicable.

### Reporting summary

Further information on research design is available in the Nature Portfolio Reporting Summary linked to this article.

## Supplementary information

Supplementary Information Supplementary Figs. 1–5.

Reporting Summary

Peer Review file

Supplementary Tables Supplementary Tables 1–18.

## Data Availability

Summary statistics are available on the PGC download page (https://pgc.unc.edu/for-researchers/download-result). Individual study data can be accessed following review and approval by the individual study cohorts; see https://pgc.unc.edu/for-researchers/individual-level-data-access/ for more information. Summary statistics for the genetic correlations are available following the procedure detailed within the relevant publications (searchable using the PMID in Supplementary Table 9) or via GWAS Catalog (https://www.ebi.ac.uk/gwas/home).

## Code availability

Analytical code is available via GitHub at: https://github.com/megskelton/gad-sympt-metagwas.
