# Supplementary material for: Genome-wide meta-analysis of quantitatively measured generalized anxiety symptoms in individuals of European ancestry
Source: Nat Hum Behav. 2026 Jun 9;10(8):1594–608. doi: 10.1038/s41562-026-02476-7 (PMC13485698; doi:10.1038/s41562-026-02476-7)
Supplement: Supplementary file 1 [file 41562_2026_2476_MOESM1_ESM.pdf]

nature human behaviour

Supplementary information https://doi.org/10.1038/s41562-026-02476-7

Genome-wide meta-analysis of quantitatively measured generalized anxiety symptoms in individuals of European ancestry

In the format provided by the authors and unedited

Supplementary Information

QQ Plot of generalised anxiety symptom severity meta-GWAS.. 1

Supplementary Figure 1 | Quantile-quantile plot of a genome-wide association meta-analysis of generalised anxiety symptom severity (N = 693,869)..................................1

MAGMA tissue expression results from FUMA...2

Supplementary Figure 2 | Gene-tissue results for a genome-wide association meta-analysis of generalised anxiety symptom severity (N = 693,869) using brain samples from BrainSpan taken at 29 different ages.. 2

Supplementary Figure 3 | Gene-tissue results for a genome-wide association meta-analysis of generalised anxiety symptom severity (N = 693,869) using brain samples from BrainSpan taken at 11 general developmental stages.. 3

Supplementary Figure 4 | Gene-tissue results for a genome-wide association meta-analysis of generalised anxiety symptom severity (N = 693,869) using 30 general tissue types from GTEx v8.. 4

Supplementary Figure 5 | Gene-tissue results for a genome-wide association meta-analysis of generalised anxiety symptom severity (N = 693,869) using 54 tissue types from GTEx v8.. 5

QQ Plot of generalised anxiety symptom severity meta-GWAS

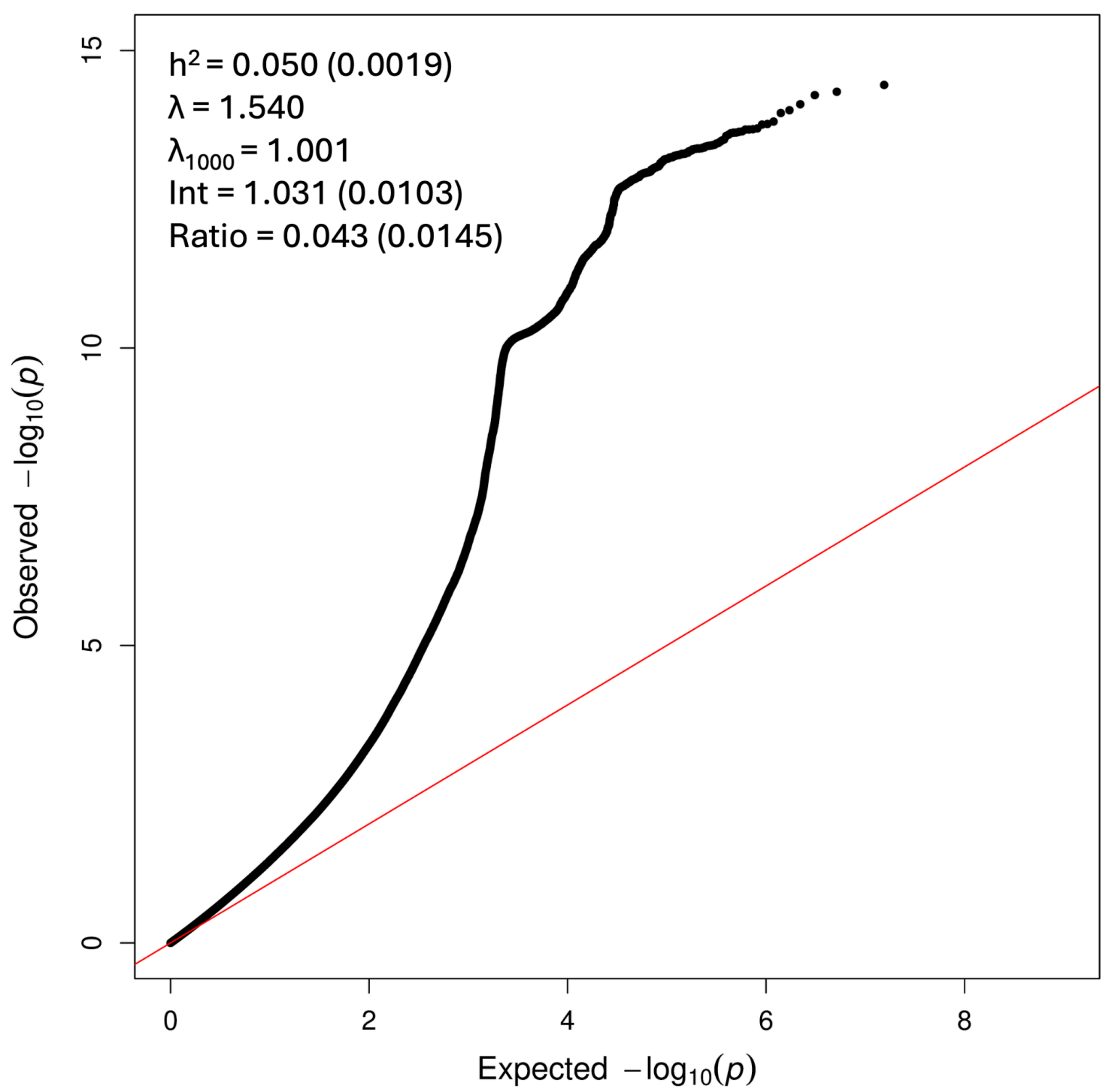

Supplementary Figure 1 | Quantile-quantile plot of a genome-wide association meta-analysis of generalised anxiety symptom severity (N = 693,869).

Note: The red line represents the expected -log10(p) under the null and the black points represent the observed -log10(p) values. Text is LDSC output, where h^2 = heritability, Int = intercept, values in brackets are standard errors of the estimates, λ = genomic inflation factor, $λ_{1000}$ = genomic inflation factor for an equivalent study with a sample size of 1000.

MAGMA tissue expression results from FUMA

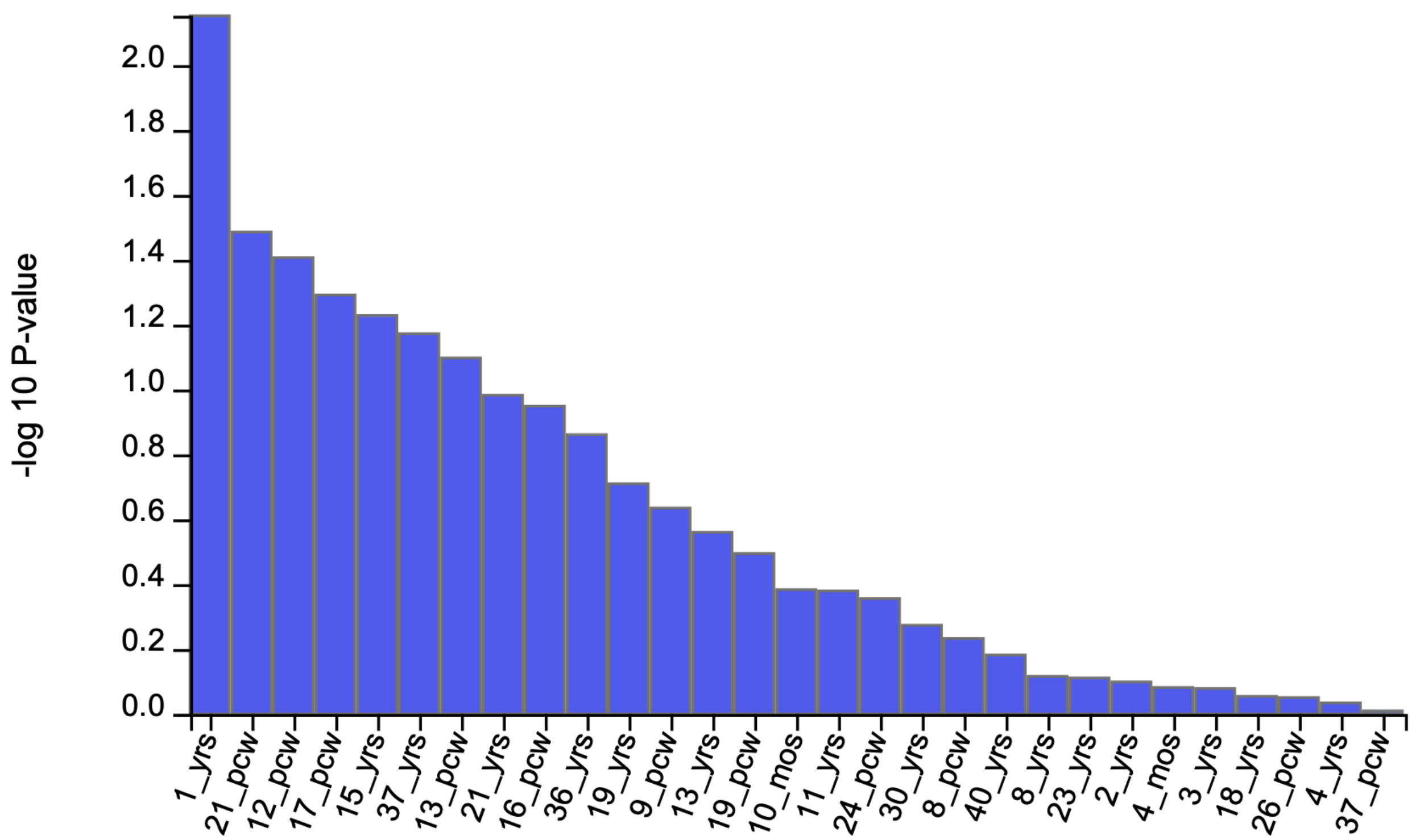

Supplementary Figure 2 | Gene-tissue results for a genome-wide association meta-analysis of generalised anxiety symptom severity (N = 693,869) using brain samples from BrainSpan taken at 29 different ages.

None passed a Bonferroni-corrected threshold for 29 tests ($p < 1.7\text{x}10^{-3}$).

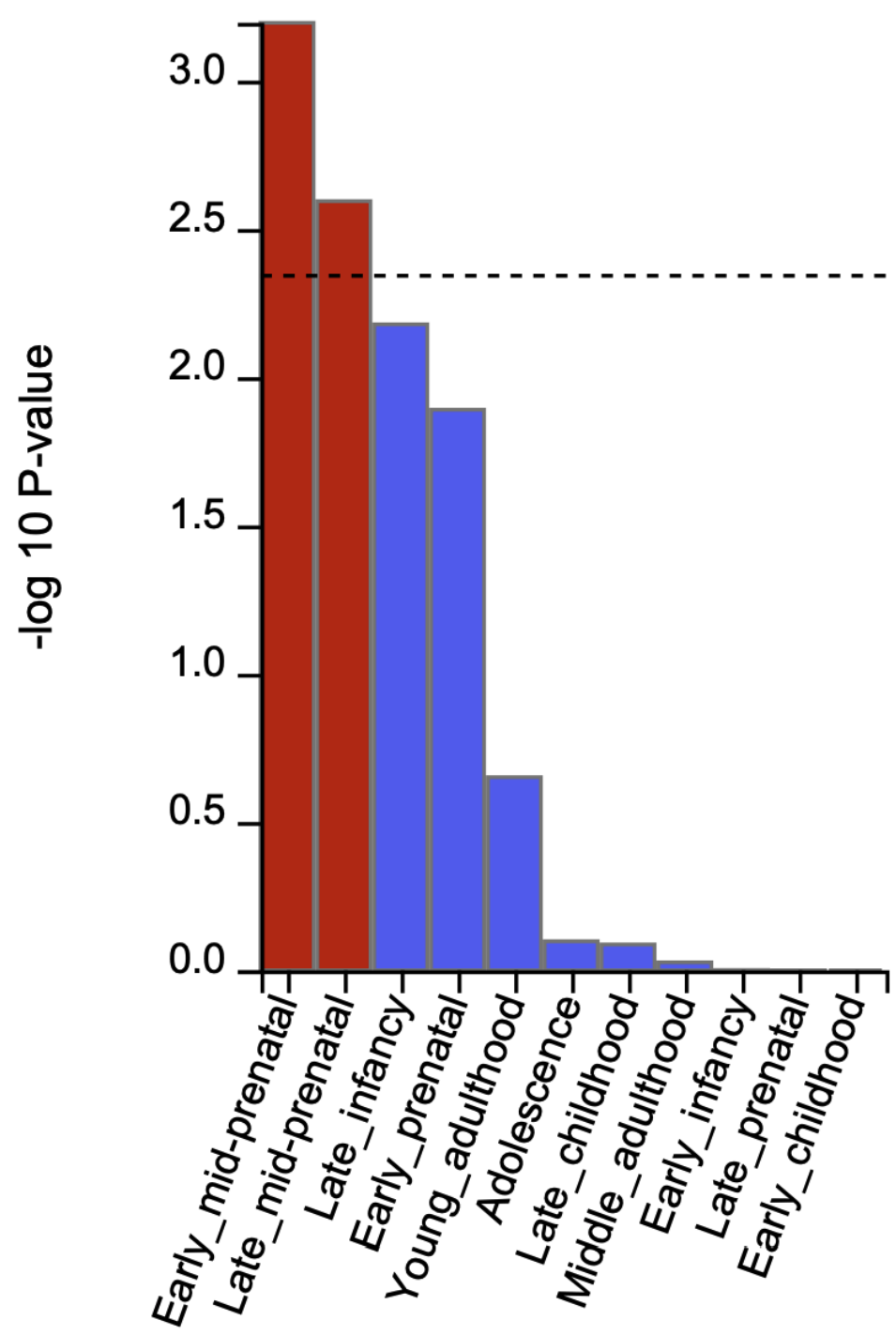

Supplementary Figure 3 | Gene-tissue results for a genome-wide association meta-analysis of generalised anxiety symptom severity (N = 693,869) using brain samples from BrainSpan taken at 11 general developmental stages.

The horizontal dotted line indicates a Bonferroni-corrected significance threshold for 11 tests ($p < 4.5 \times 10^{-3}$) and the red bars indicate tests that passed this threshold.

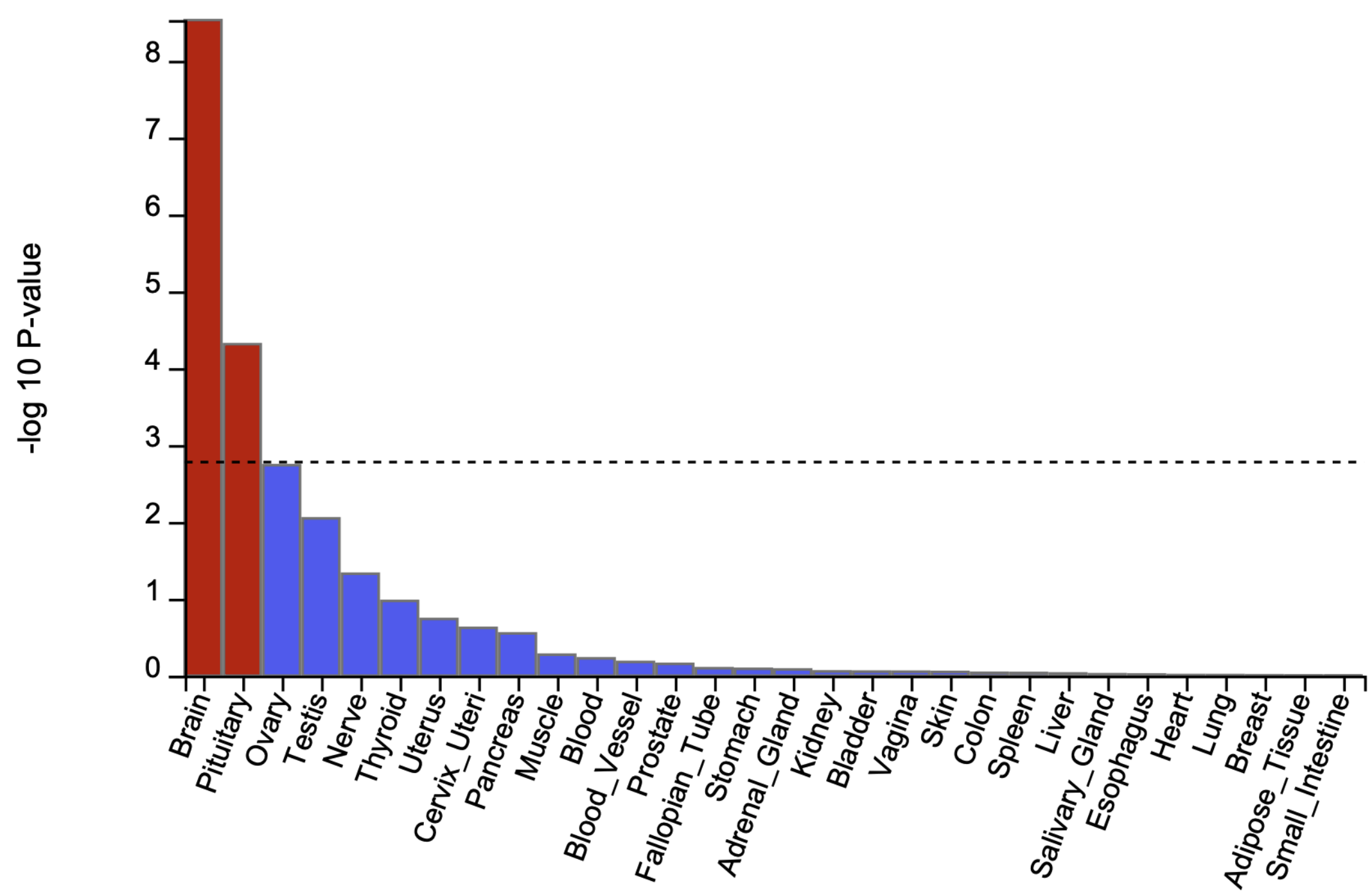

Supplementary Figure 4 | Gene-tissue results for a genome-wide association meta-analysis of generalised anxiety symptom severity (N = 693,869) using 30 general tissue types from GTEx v8. The horizontal dotted line indicates a Bonferroni-corrected significance threshold for 30 tests ($p < 1.7 \times 10^{-3}$) and the red bars indicate tests that passed this threshold.

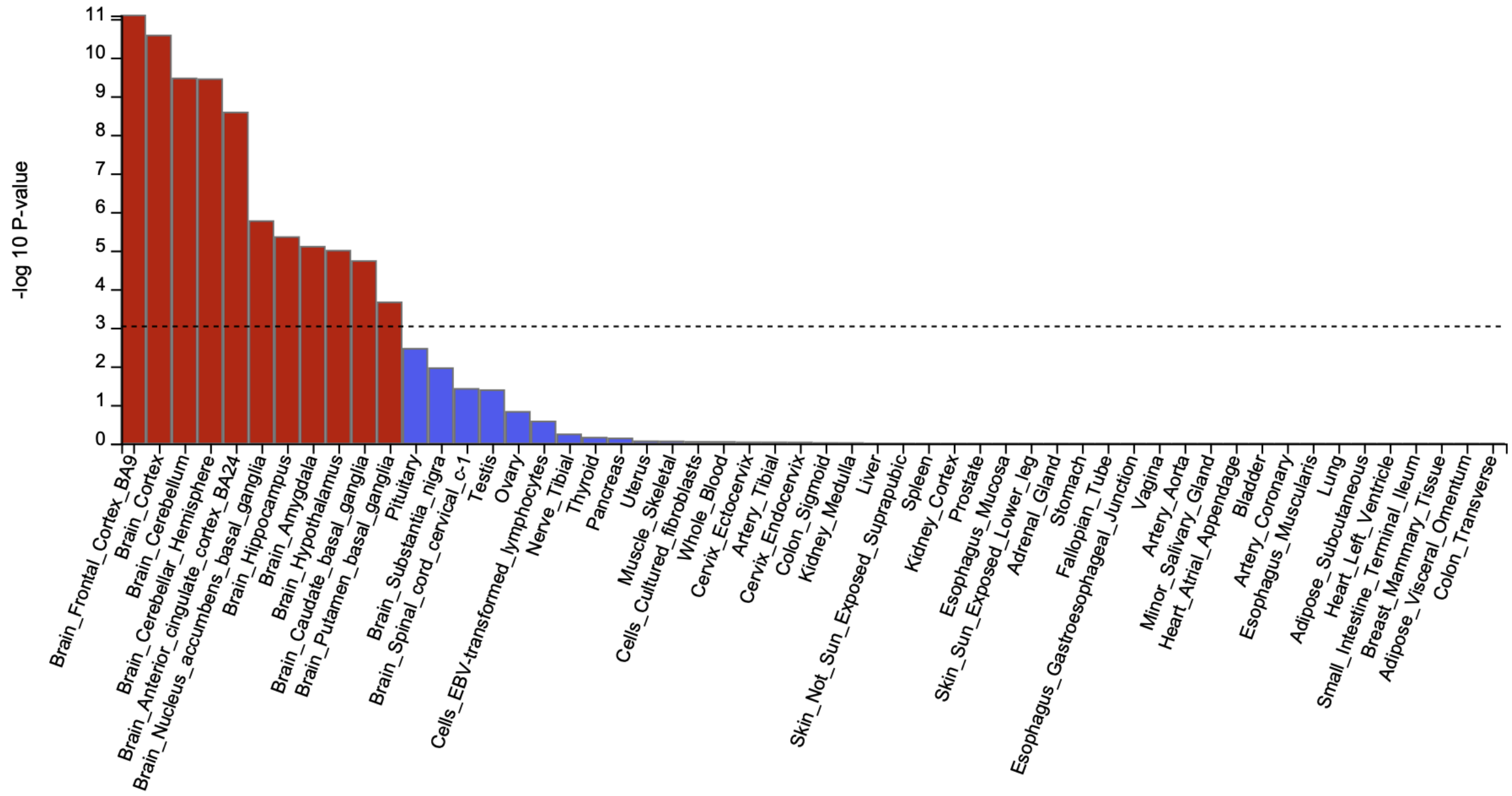

Supplementary Figure 5 | Gene-tissue results for a genome-wide association meta-analysis of generalised anxiety symptom severity (N = 693,869) using 54 tissue types from GTEx v8. The horizontal dotted line indicates a Bonferroni-corrected significance threshold for 54 tests (*p* < 9.26×10^{-4}) and the red bars indicate tests that passed this threshold. Full results are provided in Table S16.
