# Supplementary material for: Genome-wide meta-analysis of quantitatively measured generalized anxiety symptoms in individuals of European ancestry
Source: Nat Hum Behav. 2026 Jun 9;10(8):1594–608. doi: 10.1038/s41562-026-02476-7 (PMC13485698; doi:10.1038/s41562-026-02476-7)
Supplement: Supplementary file 2 [file 41562_2026_2476_MOESM2_ESM.pdf]

nature portfolio

Corresponding author(s): Thalia C Eley

Last updated by author(s): Mar 3, 2026

Reporting Summary

Nature Portfolio wishes to improve the reproducibility of the work that we publish. This form provides structure for consistency and transparency in reporting. For further information on Nature Portfolio policies, see our Editorial Policies and the Editorial Policy Checklist.

Statistics

For all statistical analyses, confirm that the following items are present in the figure legend, table legend, main text, or Methods section.

n/a	Confirmed	
☐	☒	The exact sample size (*n*) for each experimental group/condition, given as a discrete number and unit of measurement
☐	☒	A statement on whether measurements were taken from distinct samples or whether the same sample was measured repeatedly
☐	☒	The statistical test(s) used AND whether they are one- or two-sided<br>*Only common tests should be described solely by name; describe more complex techniques in the Methods section.*
☐	☒	A description of all covariates tested
☐	☒	A description of any assumptions or corrections, such as tests of normality and adjustment for multiple comparisons
☐	☒	A full description of the statistical parameters including central tendency (e.g. means) or other basic estimates (e.g. regression coefficient) AND variation (e.g. standard deviation) or associated estimates of uncertainty (e.g. confidence intervals)
☐	☒	For null hypothesis testing, the test statistic (e.g. *F*, *t*, *r*) with confidence intervals, effect sizes, degrees of freedom and *P* value noted<br>*Give P values as exact values whenever suitable.*
☒	☐	For Bayesian analysis, information on the choice of priors and Markov chain Monte Carlo settings
☒	☐	For hierarchical and complex designs, identification of the appropriate level for tests and full reporting of outcomes
☐	☒	Estimates of effect sizes (e.g. Cohen's *d*, Pearson's *r*), indicating how they were calculated

Our web collection on statistics for biologists contains articles on many of the points above.

Software and code

Policy information about availability of computer code

Data collection	No software was used for data collection for the purpose of this specific study; pre-existing data was used. The contributing cohort studies used questionnaire software such as Qualtrics, OpenClinica, and REDCAP, as detailed in their original publications.
Data analysis	This information is provided in the Methods and Supplementary Tables. Individual GWAS were performed with REGENIE (v3.1 or 3.2; https://rgcgithub.github.io/regenie/), SAIGE (v 1.3; https://saigegit.github.io/SAIGE-doc/), or PLINK (2.0; https://www.cog-genomics.org/plink/). The meta-analysis was performed in METAL (v2020-05-05; https://csg.sph.umich.edu/abecasis/metal/download/). Estimates of genetic correlations, genomic inflation factor and LD score intercept were made using LDSC (v1.0.1; https://github.com/bulik/ldsc). Clumping, SNP-level gene annotation were performed in FUMA (v1.6.5; https://fuma.ctglab.nl/). Gene-based associations, gene-set and gene-tissue expression enrichment analyses were performed in MAGMA (v1.08; https://cncr.nl/research/magma/) via FUMA. LDTrait (https://ldlink.nih.gov/?tab=ldtrait) was used to cross-reference significant loci with those from the GWAS Catalog. BEDtools (v2.31.0) was used to inspect the overlap of loci with relevant studies unavailable in the GWAS Catalog. SBayesRC (v0.2; https://github.com/zhilizheng/SBayesRC) provided an estimate of SNP-based heritability and the calculation of polygenic scores. Conditional analyses were performed with the mtCOJO tool from GCTA software (v1.94.1). Fine-mapping was performed using PolyFun (v1.0.0; https://github.com/omerwe/polyfun) and SuSiE (v0.11.92; https://stephenslab.github.io/susieR/). DrugTargetor (v1.3; https://drugtargetor.com/) was used to identify associations with individual drugs and drug classes. Data were prepared, described and visualised using R (v4; https://www.r-project.org/), the psych (https://cran.r-project.org/web/packages/psych/index.html), lavaan (https://cran.r-project.org/web/packages/lavaan/index.html) and tidyverse (https://cran.r-project.org/web/packages/tidyverse/index.html) packages.<br>Analytical code for this study is available on Github: https://github.com/megskelton/gad-sympt-metagwas.

For manuscripts utilizing custom algorithms or software that are central to the research but not yet described in published literature, software must be made available to editors and reviewers. We strongly encourage code deposition in a community repository (e.g. GitHub). See the Nature Portfolio guidelines for submitting code & software for further information.

Data

Policy information about availability of data

All manuscripts must include a data availability statement. This statement should provide the following information, where applicable:

- Accession codes, unique identifiers, or web links for publicly available datasets
- A description of any restrictions on data availability
- For clinical datasets or third party data, please ensure that the statement adheres to our policy

As per the data availability statement: Summary statistics will be made available on the PGC data-download page (https://pgc.unc.edu/for-researchers/download-result). Bona fide researchers can apply to access raw genomic data from the individual cohorts by contacting the relevant team or through the study website.

Research involving human participants, their data, or biological material

Policy information about studies with human participants or human data. See also policy information about sex, gender (identity/presentation), and sexual orientation and race, ethnicity and racism.

Reporting on sex and gender	Genetically inferred sex, based on the X chromosome, was used as part of the quality control procedure in each cohort, as is typical in GWAS. Specifically, concordance between self-reported sex and genetically inferred sex was examined, and individuals with mismatches were removed, as these can indicate genotyping errors or sample contamination. For X chromosome analyses, cohorts either stratified by genetically inferred sex or included it as a covariate. The proportion of females in each contributing sample is provided in Table S1, with the overall proportion 48%. Sex and/or gender based analyses were not performed.
Reporting on race, ethnicity, or other socially relevant groupings	We used genetically inferred ancestry estimates based on the 1000 Genomes reference panel. All participants in the main analyses were of European inferred ancestry. Polygenic risk score analysis was performed in participant samples of South Asian and African inferred ancestry to test cross-ancestry transferability of our findings. No race, ethnicity, or other socially relevant groupings were shared for this project.
Population characteristics	A total of 14 cohorts contributed to this meta-analysis and characteristics varied across each of these. Information on individual study-specific inclusion criteria is available in Table S1 e.g. lifetime experience of anxiety or depression, country population-based. Sample descriptives (mean (SD) age and percentage female) are also provided in Table S1. The total sample was 693,869 individuals, 48% female, mean age 44 years (SD = 14). Information on genotyping methods for each cohort can be found in Table S3.
Recruitment	This information is detailed for each of the 14 cohorts in Table S1. In brief, recruitment methods included online recruitment, recruitment into national biobank studies, or within clinical settings via health service providers.
Ethics oversight	Details on the ethical approval for each contributing cohort study are provided in Table S1 (e.g. "Ethical approval for the GLAD Study and NBR COPING study was obtained from the London-Fulham Research Ethics Committee (REC reference 18/LO/1218 and 20/SW/0078, respectively, COPING project no. 282754.)."). This is referred to at the start of the Methods section of the manuscript.

Note that full information on the approval of the study protocol must also be provided in the manuscript.

Field-specific reporting

Please select the one below that is the best fit for your research. If you are not sure, read the appropriate sections before making your selection.

☐ Life sciences ☒ Behavioural & social sciences ☐ Ecological, evolutionary & environmental sciences

For a reference copy of the document with all sections, see nature.com/documents/nr-reporting-summary-flat.pdf

Behavioural & social sciences study design

All studies must disclose on these points even when the disclosure is negative.

Study description	Quantitative
Research sample	Participants from established cohort studies who had previously provided generalised anxiety disorder symptom scores. This includes population-based cohort studies, studies recruiting based on a lifetime history of anxiety or depression, a population-based military veterans study, and a pregnancy-based population-based cohort study.
Sampling strategy	We identified samples with data on generalised anxiety symptom severity from communications within PGC-ANX and used as many as possible to maximise our sample size, excluding where a symptom measure had been used only in a small sample of participants as this would make subgroup measure-based secondary analyses underpowered for any meaningful conclusion. Convenience sampling was used in many cohorts, which relies on participants volunteering in response to recruitment materials, although some cohorts used population-based sampling whereby they invited all individuals within a defined population e.g. MoBa, TEDS. A power

calculation using the GCTA calculator can be found in Table S6.

Data collection	Across cohorts, symptom measures were self-report questionnaires and are detailed in Table S1. Most were provided online. Genetic data was provided via blood or saliva samples.
Timing	This varies across cohorts but broadly the start and end dates for data collection are 2007 and 2023, respectively.
Data exclusions	This varies per cohort and is detailed in the supplementary tables and original cohort publications. Quality control exclusions included missingness of genetic or phenotypic data above a specified threshold, or evidence of contamination of genetic data.
Non-participation	This is a meta-analysis of pre-existing study data and therefore no dropout occurred in respect to this specific analysis.
Randomization	All participants were analysed as a single group.

Reporting for specific materials, systems and methods

We require information from authors about some types of materials, experimental systems and methods used in many studies. Here, indicate whether each material, system or method listed is relevant to your study. If you are not sure if a list item applies to your research, read the appropriate section before selecting a response.

Materials & experimental systems

n/a	Involved in the study
☒	☐ Antibodies
☒	☐ Eukaryotic cell lines
☒	☐ Palaeontology and archaeology
☒	☐ Animals and other organisms
☒	☐ Clinical data
☒	☐ Dual use research of concern
☒	☐ Plants

Methods

n/a	Involved in the study
☒	☐ ChIP-seq
☒	☐ Flow cytometry
☒	☐ MRI-based neuroimaging

Plants

Seed stocks	N/A
Novel plant genotypes	N/A
Authentication	N/A
