# Supplementary material for: Genome-wide meta-analysis of quantitatively measured generalized anxiety symptoms in individuals of European ancestry
Source: Nat Hum Behav. 2026 Jun 9;10(8):1594–608. doi: 10.1038/s41562-026-02476-7 (PMC13485698; doi:10.1038/s41562-026-02476-7)
Supplement: Supplementary file 3 [file 41562_2026_2476_MOESM3_ESM.pdf]

nature portfolio

Peer Review File

Genome-wide meta-analysis of quantitatively measured generalized anxiety symptoms in individuals of European ancestry

Corresponding Author: Professor Thalia Eley

Version 0:

Decision Letter:

22nd August 2025

Dear Professor Eley,

Thank you once again for your manuscript, entitled "Genome-wide insights into generalised anxiety using a dimensional symptom severity approach," and for your patience during the peer review process.

Your manuscript has now been evaluated by 3 reviewers, whose comments are included at the end of this letter. Although the reviewers find your work to be of interest, they also raise some important concerns. We are very interested in the possibility of publishing your study in Nature Human Behaviour, but would like to consider your response to these concerns in the form of a revised manuscript before we make a decision on publication.

Please address all reviewer comments in full, in particular ensuring greater transparency in your methods section (and adjusting your analyses as necessary, in response to the comments of Reviewer 1).

Finally, your revised manuscript must comply fully with our editorial policies and formatting requirements. Failure to do so will result in your manuscript being returned to you, which will delay its consideration. To assist you in this process, I have attached a checklist that lists all of our requirements. If you have any questions about any of our policies or formatting, please don't hesitate to contact me.

In sum, we invite you to revise your manuscript taking into account all reviewer and editor comments. We are committed to providing a fair and constructive peer-review process. Do not hesitate to contact us if there are specific requests from the reviewers that you believe are technically impossible or unlikely to yield a meaningful outcome.

We hope to receive your revised manuscript within two months. I would be grateful if you could contact us as soon as possible if you foresee difficulties with meeting this target resubmission date.

With your revision, please:

• Include a “Response to the editors and reviewers” document detailing, point-by-point, how you addressed each editor and referee comment. If no action was taken to address a point, you must provide a compelling argument. When formatting this document, please respond to each reviewer comment individually, including the full text of the reviewer comment verbatim followed by your response to the individual point. This response will be used by the editors to evaluate your revision and sent back to the reviewers along with the revised manuscript.

• Highlight all changes made to your manuscript or provide us with a version that tracks changes.

• EXTENDED DATA FIGURES
When re-submitting your manuscript, please ensure that any supplementary figures and tables that are crucial to the manuscript’s conclusions are converted into Extended Data figures and tables to increase visibility of these data. Extended Data figures and tables are online-only (present in the online PDF and full-text HTML versions of the paper), peer-reviewed display items that provide essential background to the article but are not included in the main article due to space constraints. A maximum of ten Extended Data display items (figures and tables) is permitted.

Please use the link below to submit your revised manuscript and related files:

Link Redacted

Note: This URL links to your confidential home page and associated information about manuscripts you may have submitted, or that you are reviewing for us. If you wish to forward this email to co-authors, please delete the link to your homepage.

We look forward to seeing the revised manuscript and thank you for the opportunity to review your work. Please do not hesitate to contact me if you have any questions or would like to discuss these revisions further.

Sincerely,

[redacted]
Nature Human Behaviour

Reviewer expertise:

Reviewer #1: Psychiatric epidemiology, Genetics, GWAS meta-analysis

Reviewer #2: Psychiatric genomics, Genetics of anxiety

Reviewer #3: Psychiatric genetics, Genetics of anxiety

REVIEWER COMMENTS:

Reviewer #1 (Remarks to the Author):

The manuscript "Genome-wide insights into generalized anxiety using a dimensional symptom severity approach" reports a genome-wide association study of generalized anxiety symptoms. The study is well powered and detected several novel loci. Besides sample size, the use of the same questionnaire in most cohorts helped minimize study heterogeneity and pooling of repeated measures, when available, also strengthened the study. Overall, the study is well conducted and written, though, I would appreciate if the authors could address following comments and suggestions:

Major comments:
1. It is good to see that the authors were able to construct a PGS with predictive power in different ancestries. The manuscript reports a R2 measures, but it is not clear to me whether this refers to the R2 of the whole model or an iterative/delta R2 describing the added variance explained by the PRS on top of covariates. If it is the total model R2, then this would also reflect the variance explained by covariates, i.e. ancestry, and I would suggest to subtract the R2 explained by a baseline covariates model. If it is a R2 specific to PGS independent of covariates, I suggest to explicitly state this in the text.

2. Both GWAS and PGS analyses correct for ancestry via principal components, but I do not see a mention of sex or age. This is unusual for a psychiatric GWAS and I was wondering why these covariates were not modeled. It is unlikely that they are related to genotype, thus do not present a confounding risk, but the added explained variance may further increase the power of the study, so I wonder about the rationale of omitting these standard covariates.

3. The authors performed an inverse-variance weighted meta-analyses, which requires all measures to have the same units. Most cohorts used the GAD-7, but not all, so I wonder how the units were standardized. Was a z-score standardization or similar applied? As far as I can tell this information was only provided for the factor scores.

4. Related to 3, I suggest to somewhere explicitly state how to interpret the effect sizes/beta coefficients given the units. Something along the lines of "per G allele anxiety increased XXSD"

5. Authors write on page 16: "Heterogeneity across cohorts was assessed by inspecting the heterogeneity p-values from METAL" and then report that none passed genome-wide significance on page
6. As there are different ways to quantify/test heterogeneity, I suggest to mention the exact test. I believe here the authors refer to the Q-test. That said, I am not sure whether the reporting of genome-wide significant Q values is the most meaningful way to present heterogeneity, as lack of significant Q values could be due to lack of power of the test. Perhaps a better way to quantify heterogeneity would be to present the median I2 for nominally significant or genome-wide significant SNPs.

6. Throughout out the manuscript I suggest to also report a measure of statistical uncertainty, i.e. either standard errors or

confidence intervals, not just beta and p-value.

7. It is good to see that the X-chromosome was included, but it is not clear to me under which dosage model, i.e. whether X-chromosome inactivation was assumed or not. Were females with two effect alleles coded equivalent to one effect allele in males, assuming no escape from X-chromosome inactivation?

8. Fig S4 and S5 state “X-corrected”. Could authors elaborate what X-corrected means here?

Optional suggestions:
9. The introduction and discussion are rather heavy on methodological differences of using symptoms scores instead of binary diagnoses in GWAS. Personally, I do not think a “defense” of symptoms scores is quite necessary to this length. Shortening of these sections could leave room for discussion of new insights gained in potential neurobiological mechanisms or other discoveries.

Reviewer #2 (Remarks to the Author):

This large-scale meta-analysis investigated the genetic basis of generalized anxiety symptom severity in nearly 700,000 individuals of European ancestry. The researchers identified 82 significant genetic variants, 41 of which are new to anxiety research. Furthermore, polygenic scores derived from these findings were able to predict anxiety in diverse ancestral groups. The methodologies employed are largely standard and I do not have major criticisms. Here are some additional comments -
- The study reports very high genetic correlations (rg) between generalized anxiety symptoms, neuroticism, depression etc (0.71-0.86). These are expected, however I am curious if we can identify the unique genetic factors that are, for example, associated with anxiety symptoms only, but not depression (or neuroticism) (e.g., using methods like mtCOJO or other conditioning approaches). If extra analysis may not be possible, a discussion of it will be valuable. I think it may be more interesting if the paper can further discuss and delineate the genetic factors that are unique and shared with other anxiety-related phenotypes.
- In a similar then, it would be interesting to further discuss the novel genetic loci discovered that have not been reported in MDD or other studies of anxiety related phenotypes. The two discussed loci seem to have been reported in some related studies in other studies?
- The primary meta-analysis was conducted exclusively in individuals of European ancestry. While the PGS was tested in other groups, the discovery of the 82 loci themselves is biased toward this single population. Please further discuss this limitation, and please see if any comparisons can be made to existing anxiety-related phenotype gwas in the non-European populations.
- The 5.9% estimate for common variants is notably low. Besides that the anxiety symptoms may fluctuate as admitted in their limitations, what are the other possible explanations? How does this estimate compare with the snp-heritability estimates of other related traits, eg self-reported depressive symptoms/anxiety symptoms etc?
- Please also further discuss the possible consequences and the degree of heterogeneity across the study cohorts. In addition, were any cohorts enriched for individuals with clinical anxiety disorders? If so, could this affect the generalizability of findings?
- Are there any plans to analyze the genetics of individual domain of anxiety symptoms? Please kindly discuss.

Reviewer #3 (Remarks to the Author):

Reviewer comments on the manuscript titled "Genome-wide insights into generalised anxiety unsing a dimensional symptom severity approach", by Skelton et al, submitted for publication in NHB.

This is an impressive analysis of nearly 700K individuals focused on the identification of variants associated with anxiety, one of the most prevalent mental health issues of current times.

The paper is remarkably well written, and the emphasis on continuous measures is commendable. The authors opted to use rigorous and well-established methods as opposed to novel approaches of unknown validity, which is also a plus.

The authors identify dozens of loci, many of which replicating previous findings from the PGC-Anxiety consortium, while noting some sample overlap. SNP-based heritability was remarkably low, which is not surprising. PRS generalizability was modest, but also not unexpected.

Implicated pathways from the ontology terms as reported are somewhat vague: "postsynaptic membrane", "synaptic membrane", "axon". These don't provide much insight, so it's a gap that needs to be filled, perhaps by future studies. Results from the analysis for targets was also somewhat disappointing, but are nonetheless merited for the inquiry and, in my view, should be seen by colleagues working in this area, not left in the drawer.

I only have one minor comment: It would be good to add a sentence or two at the beginning of the methods describing the overall administrative aspects of the study. It's a meta-analysis, but the investigators of each of the studies presumably had

to run consistent analyses, which then were later pooled together. Using the terminology from Zugman et al (ref below), this would be a "meta-analysis with access to indvidual participant data", as opposed to the more typical meta-analyses that use results from disparate published studies. Is that so? The reader needs to put together pieces that are left implied throughout the Methods and Results sections. It would help to provide a better understanding of the study as a whole.

Zugman A et al. Mega-analysis methods in ENIGMA: The experience of the generalized anxiety disorder working group. Hum Brain Mapp. 2022 Jan;43(1):255-277. doi: 10.1002/hbm.25096. PMID: 32596977; PMCID: PMC8675407.

Version 1:

Decision Letter:

Our ref: NATHUMBEHAV-25073118A

6th February 2026

Dear Dr. Eley,

Thank you for submitting your revised manuscript "Genome-wide analyses of quantitative generalised anxiety symptom severity" (NATHUMBEHAV-25073118A). It has now been seen by the original referees and their comments are below. As you can see, the reviewers find that the paper has improved in revision. We will therefore be happy in principle to publish it in Nature Human Behaviour, pending minor revisions to satisfy the referees' final requests and to comply with our editorial and formatting guidelines.

We are now performing detailed checks on your paper and will send you a checklist detailing our editorial and formatting requirements within two weeks. Please do not upload the final materials and make any revisions until you receive this additional information from us.

Please do not hesitate to contact me if you have any questions.

Sincerely,

[redacted]
Nature Human Behaviour

Reviewer #1 (Remarks to the Author):

The authors have substantially improved the manuscript by clarifying the methods, improving the evaluation of heterogeneity and improving the X-chromosome analyses. I thank the authors for their thorough response and do not have further comments.

Reviewer #2 (Remarks to the Author):

My comments have been adequately addressed and I appreciate authors for carrying out additional analysis. I have no further major comments.

Reviewer #3 (Remarks to the Author):

The manuscript is good in my view. My previous review indicated that and I overall remain satisfied with the work. In my opinion, the authors address satisfactorily most of the comments from the other reviewers as well, and have thought about those perceived issues (which I see as minor).

Open Access This Peer Review File is licensed under a Creative Commons Attribution 4.0 International License, which permits use, sharing, adaptation, distribution and reproduction in any medium or format, as long as you give appropriate credit to the original author(s) and the source, provide a link to the Creative Commons license, and indicate if changes were made.
In cases where reviewers are anonymous, credit should be given to 'Anonymous Referee' and the source.
The images or other third party material in this Peer Review File are included in the article's Creative Commons license, unless indicated otherwise in a credit line to the material. If material is not included in the article's Creative Commons license and your intended use is not permitted by statutory regulation or exceeds the permitted use, you will need to obtain permission directly from the copyright holder.
To view a copy of this license, visit https://creativecommons.org/licenses/by/4.0/

Response to the editors and reviewers

[redacted] and reviewers,

Thank you for the consideration and review of our manuscript, “Genome-wide insights into generalised anxiety using a dimensional symptom severity approach”. We have revised the manuscript to address the reviewers’ comments and provided detailed point-by-point responses. The reviewers’ comments are in black font and our responses in blue font, with excerpts from the revised manuscript shown as indented, italicised text. We have also included a tracked-changes version of the revised Word document.

During the revision process, we identified an error that led us to re-run the analyses. As a result, the final sample size decreased from 696,563 to 693,869, and the number of genome-wide significant loci from 76 to 74. The number of novel loci mirrored this reduction, from 41 to 39. Genetic correlation and heritability estimates remain the same. Other results, including finemapping and gene-based associations, are largely consistent with the original submission; minor changes to gene-based results are attributable to the re-analysis of the X-chromosome, as requested by Reviewer 1. We have also revised the title, replacing ‘dimensional’ with the more field-appropriate ‘quantitative’, such that it is now “Genome-wide analyses of quantitative generalised anxiety symptom severity”.

We believe these revisions have strengthened the manuscript and hope that we have adequately addressed the reviewers' concerns. We greatly appreciate the opportunity to revise our work and we look forward to hearing from you.

REVIEWER COMMENTS:

Reviewer #1 (Remarks to the Author):

The manuscript “Genome-wide insights into generalized anxiety using a dimensional symptom severity approach” reports a genome-wide association study of generalized anxiety symptoms. The study is well powered and detected several novel loci. Besides sample size, the use of the same questionnaire in most cohorts helped minimize study heterogeneity and pooling of repeated measures, when available, also strengthened the study. Overall, the study is well conducted and written, though, I would appreciate if the authors could address following comments and suggestions:

Major comments:
1. It is good to see that the authors were able to construct a PGS with predictive power in different ancestries. The manuscript reports a R2 measures, but it is not clear to me whether

this refers to the R2 of the whole model or an iterative/delta R2 describing the added variance explained by the PRS on top of covariates. If it is the total model R2, then this would also reflect the variance explained by covariates, i.e. ancestry, and I would suggest to subtract the R2 explained by a baseline covariates model. If it is a R2 specific to PGS independent of covariates, I suggest to explicitly state this in the text.

We thank the reviewer for pointing out the ambiguity in our description of the R^2 measure. We confirm that the reported values reflect the variance explained by the PRS above and beyond the covariates. To make this explicit, we have revised the Methods section (p.20) to state:

> *"The variance explained by the PRS was calculated as the difference in R^2 between a full model including the PRS and covariates, and a null model with only covariates."*

And the results (p.9):

> "*These R^2 values represent the variance explained by the PRS beyond that accounted for by covariates.*"

2. Both GWAS and PGS analyses correct for ancestry via principal components, but I do not see a mention of sex or age. This is unusual for a psychiatric GWAS and I was wondering why these covariates were not modeled. It is unlikely that they are related to genotype, thus do not present a confounding risk, but the added explained variance may further increase the power of the study, so I wonder about the rationale of omitting these standard covariates.

Thank you for drawing attention to this point. While age and sex have often been included as covariates in psychiatric GWAS, there has been a shift away from this practice in recent years (e.g., PGC-MDD 2025 https://www.cell.com/cell/fulltext/S0092-8674(24)01415-6).

Our rationale for omitting them is twofold. First, as the reviewer notes, age and sex are not associated with genotype and therefore cannot act as confounders in SNP effect estimation; adjusting for them is not required to obtain unbiased effect estimates. Second, the influences of sex and age on psychiatric phenotypes are complex and likely reflect genuine effect moderation rather than nuisance variance. We therefore consider these variables of interest in their own right, warranting dedicated follow-up analyses rather than treating them solely as nuisance variance to be partialled out. While including them as covariates might have modestly increased power, assessing this would require re-analysis across all contributing cohorts. We have added the following clarification to the Methods (p.18) to make our rationale transparent:

"*We did not include age or sex as covariates, as they are not confounders of genetic effects and may represent effect moderators of interest warranting dedicated follow-up investigation, rather than variables to be adjusted for.*"

3. The authors performed an inverse-variance weighted meta-analyses, which requires all measures to have the same units. Most cohorts used the GAD-7, but not all, so I wonder how

the units were standardized. Was a z-score standardization or similar applied? As far as I can tell this information was only provided for the factor scores.

All scores were standardised to have a mean of zero and a standard deviation of one. To clarify this, we have added a sentence to the Methods (p.17):

> "*All scores, whether single timepoint, mean or factor score, were standardised to have a mean of zero and a standard deviation of one*."

4. Related to 3, I suggest to somewhere explicitly state how to interpret the effect sizes/beta coefficients given the units. Something along the lines of "per G allele anxiety increased XXSD"

Thank you for this helpful suggestion. To aid interpretation, we have added the following sentence to the Methods (p.18):

> "*The beta values from the meta-analysis represent the associated change in standard deviation units of generalised anxiety symptom score per additional copy of the effect allele*."

We have also added a footnote to Supplementary Table 4:

> "*Note: As an example, for rs6684464, the beta is -0.012. Therefore, each additional copy of the A1 allele (in this instance, T), is associated with a 0.012 SD decrease in generalised anxiety symptom score*."

5. Authors write on page 16: "Heterogeneity across cohorts was assessed by inspecting the heterogeneity p-values from METAL" and then report that none passed genome-wide significance on page 6. As there are different ways to quantify/test heterogeneity, I suggest to mention the exact test. I believe here the authors refer to the Q-test. That said, I am not sure whether the reporting of genome-wide significant Q values is the most meaningful way to present heterogeneity, as lack of significant Q values could be due to lack of power of the test. Perhaps a better way to quantify heterogeneity would be to present the median I2 for nominally significant or genome-wide significant SNPs.

Thank you for this suggestion. We confirm that heterogeneity was assessed using the p-value from Cochran's Q-test as implemented in METAL. In line with the reviewer's recommendation, we now also report the median I^2 values across cohorts for all genome-wide significant SNPs and for independent lead SNPs. The relevant Methods (p.18) section now reads:

> "*Heterogeneity across cohorts was assessed by inspecting the p-values from Cochran's Q-test as implemented in METAL. In addition, we calculated the median I^2 values (HetISq in METAL) for SNPs reaching genome-wide significance ($p < 5x10^{-8}$) and for independent lead SNPs*."

The Results section (p.6) has also been revised accordingly:

> "*Among the 6,012 genome-wide significant SNPs, study heterogeneity accounted for a moderate proportion of variance in effect size estimates, with a median I^2 across cohorts*

of 29.7%. In contrast, the median I^2 for the 80 independent genome-wide significant SNPs was 0%, reflecting highly consistent effects across cohorts. Heterogeneity statistics for the lead SNPs are presented in Table S4."

6. Throughout out the manuscript I suggest to also report a measure of statistical uncertainty, i.e. either standard errors or confidence intervals, not just beta and p-value.

In response to the reviewer's suggestion, we have added standard errors to the lead SNPs reported in the results (p.5):

> "*The top signal came from an intergenic locus near the long noncoding RNA RP4-598G3.1 on chromosome 1 (lead SNP rs7546305, $p = 3.8 \times 10^{-15}$, beta = -0.014, SE = 0.0017), followed by a locus within an intron of PCLO on chromosome 7 (lead SNP rs1476548, $p = 4.9 \times 10^{-15}$, beta = -0.014, SE = 0.0018)*."

Standard errors are provided for subgroup genetic correlations, SNP-based heritability, and the LDSC intercept. For external genetic correlations, the 95% confidence intervals are shown in Figure 2 and standard errors are provided in Supplementary Table 9. We did not duplicate these values in the main text in order to maintain readability where we have reported ranges of genetic correlations for groups of traits, but they are visible in the accompanying figure and table. Finally, we added beta estimates and their standard errors to the PRS results (p.9), for example:

> "*The PRS significantly explained 2.9% of the variance (R^2) in generalised anxiety symptom scores in an independent European ancestry sample (beta = 0.55, SE = 0.054, $p = 8.9 \times 10^{-24}$)...*".

7. It is good to see that the X-chromosome was included, but it is not clear to me under which dosage model, i.e. whether X-chromosome inactivation was assumed or not. Were females with two effect alleles coded equivalent to one effect allele in males, assuming no escape from X-chromosome inactivation?

Individual cohorts used either PLINK2 or REGENIE for their GWAS, including analysis of the X-chromosome where available. Neither method assumes X-chromosome inactivation, and male genotypes are coded as diploid-scaled (0/2) by default. PLINK 1.9 was used by two cohorts (n = 4,657), for which male genotypes are coded as haploid-scaled by default. We have removed those for this resubmission but were able to add X-chromosome analysis from AGDS (n = 7,689). Sex was included as a covariate in the X-chromosome analyses to adjust for baseline differences between males and females. Nonetheless, as noted in our response to comment (2), more focused future studies are required to explore potential sex-specific effects and alternative dosage models.

There were no significant hits in the X-chromosome. Most downstream analyses including LDSC, finemapping, and PRS calculation, are autosome-based and as such this change does not affect those results. Gene-based analyses using MAGMA, including DrugTargetor, were

impacted minimally: the number of gene sets passing the significance threshold rose from three to six; gene-tissue results were the same besides minor p-values changes; there was one additional significant drug class association from Drug Targetor. We have clarified our approach in the Methods (p.18):

> "*X-chromosome data were analysed from seven cohorts (166,852 variants), with male genotypes coded as diploid (0/2) and sex included as a covariate.*"

8. Fig S4 and S5 state "X-corrected". Could authors elaborate what X-corrected means here?

We thank the reviewer for drawing attention to this. The label "X-corrected" was an inadvertent placeholder, and we have now replaced it with the correct term "Bonferroni-corrected" in Figures S4 and S5.

Optional suggestions:
9. The introduction and discussion are rather heavy on methodological differences of using symptoms scores instead of binary diagnoses in GWAS. Personally, I do not think a "defense" of symptoms scores is quite necessary to this length. Shortening of these sections could leave room for discussion of new insights gained in potential neurobiological mechanisms or other discoveries.
Thank you for this thoughtful suggestion. While we appreciate the perspective that a more concise treatment of the methodological considerations could suffice, we have opted to retain the current level of detail. Our reasoning is that Reviewer 3 noted that the manuscript was particularly well written, and we therefore believe the existing balance between methodological context and discussion of findings remains appropriate.

Reviewer #2 (Remarks to the Author):

This large-scale meta-analysis investigated the genetic basis of generalized anxiety symptom severity in nearly 700,000 individuals of European ancestry. The researchers identified 82 significant genetic variants, 41 of which are new to anxiety research. Furthermore, polygenic scores derived from these findings were able to predict anxiety in diverse ancestral groups. The methodologies employed are largely standard and I do not have major criticisms. Here are some additional comments -

1) The study reports very high genetic correlations (rg) between generalized anxiety symptoms, neuroticism, depression etc (0.71-0.86). These are expected, however I am curious if we can identify the unique genetic factors that are, for example, associated with anxiety symptoms only, but not depression (or neuroticism) (e.g., using methods like mtCOJO or other conditioning approaches). If extra analysis may not be possible, a discussion of it will be valuable. I think it may be more interesting if the paper can further

discuss and delineate the genetic factors that are unique and shared with other anxiety-related phenotypes.

We agree that this is a highly relevant and interesting question, although conditional analyses present a particular challenge due to the statistical power required and the unstable effect estimates that can arise when traits are very highly correlated. For example, the PGC Cross-Disorder working group recently demonstrated in a preprint that current GWASs of major depression, anxiety disorders, and PTSD cannot be meaningfully separated in conditional analyses (https://doi.org/10.1101/2025.01.14.25320574). Nonetheless, we ran mtCOJO in response to the reviewer's suggestion, conditioning our generalised anxiety symptoms meta-analysis on case-control depression, depression symptoms, neuroticism, and case-control anxiety. All had sufficient instruments to run the analysis except depression symptoms. We additionally quantified the impact of conditioning on SNP-heritability using LDSC and block jackknifing. We have added the following to the Methods (p.20)

> *"High genetic correlations, such as those often observed between anxiety, depression, and neuroticism, do not necessarily indicate identical biology; even when most loci are shared, traits may involve different biological pathways, tissue enrichments, or show individual patterns of relationships with other traits. Identifying unique genetic influences on anxiety is important to better understand its specific aetiology and inform potential treatment pathways. However, conditional analyses, especially in the presence of strong genetic correlations, are statistically challenging and require substantial power, and therefore should be interpreted cautiously. We ran mtCOJO[34], which performs conditional analyses between summary statistics to provide marginal effect estimates for the trait of interest. We conditioned our anxiety meta-analysis on depression diagnosis[80] (98 index SNPs), neuroticism[84] (67 index SNPs) and anxiety diagnosis[23] (11 index SNPs). Depression symptoms[85] was underpowered (2 index SNPs) for the analysis. We also estimated the SNP-heritability of each set of conditioned summary statistics with LDSC and used block jack-knifing to compare these to the unconditioned heritability estimate."*

As well as this addition to the Results (p.9):

> *"To assess whether genome-wide significant loci for generalised anxiety symptoms represented effects unique to this phenotype, we performed multi-trait conditional and joint analysis (mtCOJO[34]), conditioning on highly genetically correlated traits. Conditioning on neuroticism (67 instruments, GSMR $b_{xy} = 0.47 \pm 0.02$) resulted in 9 remaining genome-wide significant SNPs across 2 loci (Table S10). Conditioning on case-control anxiety (11 instruments, $b_{xy} = 0.22 \pm 0.02$) yielded 66 significant SNPs across 6 loci, and on depression (98 instruments, $b_{xy} = 0.31 \pm 0.01$) produced 76 SNPs across 6 loci. Approximately half of SNP effect sizes attenuated, and standard errors had a median inflation of 7-9%, suggesting the loss of significance was not solely attributable to reduced power. Sign changes were observed in 17-20% of variants for each trait, consistent with instability as expected under high genetic correlations. The LDSC SNP-heritability estimates of the conditioned summary statistics were significant (neuroticism $h^2 = 0.014$, $SE = 0.001$, $p = 1.02x10^{-42}$; case-control anxiety $h^2 = 0.018$, $SE = 0.0011$, $p = 1.54x10^{-59}$; case-control depression $h^2 = 0.017$, $SE = 0.0011$, $p = 7.34x10^{-51}$), but block*

jack-knifing confirmed they were significantly lower than the original estimate ($Z = 33.24$, $p = 3.3x10^{-242}$; $Z = 28.61$, $p = 5.0x10^{-180}$; $Z = 30.48$, $4.24x10^{-204}$, respectively)."

And the following to the Discussion (p.13):

"*A strong genetic correlation was observed with neuroticism, a well-established risk factor for anxiety[56]. This likely reflects both genuine shared liability and conceptual or item-level overlap between the measures. Conditional analyses indicated extensive overlap between loci associated with generalised anxiety symptoms and those implicated in neuroticism, case-control anxiety and depression. Many genome-wide significant loci may therefore reflect a broader neuroticism-related liability, although the presence of anxiety-specific effects cannot be excluded due to the statistical noise introduced by conditioning on highly genetically correlated traits."*

2) In a similar then, it would be interesting to further discuss the novel genetic loci discovered that have not been reported in MDD or other studies of anxiety related phenotypes. The two discussed loci seem to have been reported in some related studies in other studies?

We thank the reviewer for this helpful suggestion. We have clarified that the top two loci have previously been implicated in internalising GWAS to avoid the impression that they were novel (p.5):

"These loci have both previously been associated with internalising traits, including anxiety (Table S5)."

In addition to these, our meta-analysis identified 16 loci not reported in prior GWAS of anxiety, depression, or neuroticism. For example, rs58179213 (annotated to REEP4, HR, FAM160B2, DMTN, FAM160B2, NUDT18, LGI3, SFTPC, BMP1) and rs4886369 (no gene annotations but a single prior association with verbal short-term memory in GWAS Catalog) show no prior associations with internalising traits in the GWAS Catalog. These loci do not currently point to obvious functional pathways related to anxiety but highlight new avenues for exploration. We have added the following text to the Results (p.10):

"In contrast, gene annotations for loci novel for associations with internalising traits did not indicate clear neurobiological pathways implicated in anxiety. For example, rs58179213 is proximal to multiple genes with evidence for involvement in diverse processes, including lung function (SFTPC), intestinal barrier function and immunoprotective inflammation (FHIP2B), and hair growth (HR). While such functions may influence anxiety via indirect pathways (e.g. gut-brain axis, somatic anxiety symptoms), replication of these loci is required to provide the greater power necessary to determine their relevance."

3) The primary meta-analysis was conducted exclusively in individuals of European ancestry. While the PGS was tested in other groups, the discovery of the 82 loci

themselves is biased toward this single population. Please further discuss this limitation, and please see if any comparisons can be made to existing anxiety-related phenotype gwas in the non-European populations.

We thank the reviewer for highlighting this important point. Our meta-analysis was conducted in European ancestry cohorts, which limits the direct generalisability of the significant loci and downstream findings to other populations. While our polygenic score analyses demonstrated cross-ancestry prediction in African and South Asian samples, ancestry-specific GWAS will be needed to fully characterise the genetic architecture of anxiety in non-European groups and ensure health equity in the context of using genetic information to aid health decision making. To our knowledge, the only existing genome-wide significant association found in non-European populations is from the Levey et al. (2020) analysis in the MVP cohort, which found one hit; an insertion variant that was not present in our meta-analysis results, likely as it is rare (below 1% MAF) outside of African ancestry populations.

We have added text to the Discussion (p.14) to make this limitation explicit:

> "*A key limitation of the present meta-analysis is its restriction to cohorts of European ancestry, due to lack of data for other ancestries at sufficient scale for GWAS analysis. Our PRS findings support a degree of shared genetic influence with African and South Asian populations. Ancestry-specific modelling across diverse populations remains necessary to identify population-specific risk loci, such as that identified in a previous analysis of African-American participants[26], and ensure equitable benefits from genetic discoveries.*"

4) The 5.9% estimate for common variants is notably low. Besides that the anxiety symptoms may fluctuate as admitted in their limitations, what are the other possible explanations? How does this estimate compare with the snp-heritability estimates of other related traits, eg self-reported depressive symptoms/anxiety symptoms etc?

Although lower than, for example, the recent PGC case-control analysis of anxiety (10.1%) and of depression (8.4%), our finding is consistent with previous analyses of self-reported symptom scores. For example, the GAD-2 measure in MVP (Levey et al., 2020) yielded a SNP-based heritability estimate of 5.6%, and an analysis of depression symptoms reported around 4% SNP h^2 (Direk et al., 2017). While our sample size is larger than these studies, we included a greater number of cohorts, which may have introduced heterogeneity from various sources. For example, we would expect to see lower estimates of heritability compared to studies using the UK Biobank, because the UK Biobank is homogeneous in terms of genotyping method, QC, age, sex, geographic location, and socioeconomic status. Single-sample studies with access to individual-level data can also use methods such as GCTA which can yield higher heritability estimates than the summary statistic-based methods we used. We have incorporated a more detailed discussion of this into the following section of the Discussion (p.12-13):

> "*Our SNP-based heritability estimate (5.9%) aligned with an existing GWAS of GAD symptom severity (5.6%)[26] while remaining lower than liability scale estimates from case-*

control anxiety meta-analyses[23]. In contrast to traditional case-control phenotyping, which aims to maximise clinical specificity through diagnostic thresholds, our approach leveraged the full spectrum of symptom variability, increasing power for discovery[52] and capturing genetic risk relevant to both subclinical and clinical presentations. The key differences between clinical diagnoses and symptom severity measures relate to the presence of distress and impairment, and symptom duration. While subclinical symptoms can still cause distress and impairment, this is not always true for lower levels of anxiety severity, potentially contributing to some diagnosis-specific genetic variance. Similarly, diagnostic tools often assess lifetime occurrence and require symptoms to be present for a minimum period of time (six months for GAD), whereas symptom severity scales typically capture recent experiences (e.g., past two weeks), introducing greater susceptibility to transient fluctuations and measurement noise. Consistent with this, GWAS of depression symptom severity typically yield lower SNP-based heritability estimates than case-control analyses[53–55]. We aimed to partially address and control for temporal fluctuations and better approximate a stable underlying trait[16] by incorporating assessments from multiple timepoints into our analysis, where available. In addition, our meta-analysis combined data from multiple cohorts with differences in phenotype definitions, genotyping arrays, imputation methods, quality control procedures, and population structure adjustments, which may have introduced further heterogeneity and reduced the observed SNP-based heritability. By contrast, single-cohort studies with individual-level data are more homogeneous and can implement alternatives to summary-statistics based methods. Despite our lower heritability estimate, the strong genetic correlation observed between our phenotype and case-control anxiety suggests that GAD symptom severity captures much of the same genetic risk. This finding is consistent with a recent analysis of obsessive-compulsive symptoms[56]."

5) Please also further discuss the possible consequences and the degree of heterogeneity across the study cohorts. In addition, were any cohorts enriched for individuals with clinical anxiety disorders? If so, could this affect the generalizability of findings?

We thank the reviewer for this important point. As noted in the manuscript, our study included both population-based and clinical cohorts enriched for anxiety disorders, which introduces some heterogeneity in symptom distributions. Unfortunately, our subgroup analyses were underpowered to estimate genetic correlations between cohorts ascertained under these two methods. Clinical cohorts are enriched for individuals with more severe symptoms (although not uniformly as they typically represent 'lifetime' diagnosis which may presently be in remission, while symptom measures cover recent time periods), which could influence effect size estimates and limit generalisability to broader populations. However, this overrepresentation of higher severity symptoms, relative to the population, was necessary to better represent the full phenotypic spectrum and thereby boost power to detect genetic effects. We have clarified these points in the Discussion (p.14-15) to highlight the potential consequences of cohort heterogeneity and ascertainment differences.

"*Our subgroup analyses based on measure and ascertainment method were largely underpowered to reliably estimate SNP-based heritability or correlations. Although sufficiently powered comparisons indicated high genetic overlap, we cannot be certain that all cohorts captured the same underlying genetic architecture. While population-based cohorts allow assessment of the full range of symptoms, symptom severity measures typically better distinguish variation at the upper end of the distribution. This results in highly skewed symptom severity scores, as most participants report few or no symptoms, whereas individuals in clinical cohorts typically report more symptoms. Combining population-based cohorts with studies selecting on diagnosis introduces some heterogeneity and may limit generalisability to broader populations, but can help yield a more normally distributed phenotype for GWAS analysis. This combination may have improved statistical power for detecting associations in our study*."

6) Are there any plans to analyze the genetics of individual domain of anxiety symptoms? Please kindly discuss.

We thank the reviewer for this suggestion. Investigating the genetics of individual anxiety symptom domains, such as cognitive (e.g., worry) versus physiological (e.g., restlessness) symptoms, is indeed of high interest. However, this level of phenotypic granularity is beyond the scope of the present study. These domain-specific analyses should be addressed in future work, and we have added this to the future direction section of the Discussion (p.14):

"*Expanding future studies to incorporate a broader range of anxiety symptom measures will enable more robust, transdiagnostic translation of findings. Furthermore, future work could examine the genetic architecture of individual symptom domains, such as cognitive versus physiological symptoms, to better understand the biological specificity of these.*"

Reviewer #3 (Remarks to the Author):

Reviewer comments on the manuscript titled "Genome-wide insights into generalised anxiety unsing a dimensional symptom severity approach", by Skelton et al, submitted for publication in NHB.

This is an impressive analysis of nearly 700K individuals focused on the identification of variants associated with anxiety, one of the most prevalent mental health issues of current times.

The paper is remarkably well written, and the emphasis on continuous measures is commendable. The authors opted to use rigorous and well-established methods as opposed to novel approaches of unknown validity, which is also a plus.

The authors identify dozens of loci, many of which replicating previous findings from the PGC-Anxiety consortium, while noting some sample overlap. SNP-based heritability was remarkably low, which is not surprising. PRS generalizability was modest, but also not unexpected.

Implicated pathways from the ontology terms as reported are somewhat vague: "postsynaptic membrane", "synaptic membrane", "axon". These don't provide much insight, so it's a gap that needs to be filled, perhaps by future studies. Results from the analysis for targets was also somewhat disappointing, but are nonetheless merited for the inquiry and, in my view, should be seen by colleagues working in this area, not left in the drawer.

I only have one minor comment: It would be good to add a sentence or two at the beginning of the methods describing the overall administrative aspects of the study. It's a meta-analysis, but the investigators of each of the studies presumably had to run consistent analyses, which then were later pooled together. Using the terminology from Zugman et al (ref below), this would be a "meta-analysis with access to indvidual participant data", as opposed to the more typical meta-analyses that use results from disparate published studies. Is that so? The reader needs to put together pieces that are left implied throughout the Methods and Results sections. It would help to provide a better understanding of the study as a whole.

Zugman A et al. Mega-analysis methods in ENIGMA: The experience of the generalized anxiety disorder working group. Hum Brain Mapp. 2022 Jan;43(1):255-277. doi: 10.1002/hbm.25096. PMID: 32596977; PMCID: PMC8675407.

We thank the reviewer for their generous comments. We agree that some aspects, such as the drug target analyses, yielded limited findings, but we hope that this work helps pave the way for future anxiety genetics research with larger samples and more in-depth methods to focus on these areas. We appreciate the suggestion to clarify the overall study design. As the reviewer correctly inferred, this was a meta-analysis with each contributing cohort - besides the MVP sumstats - having access to individual participant data, with which they conducted GWAS specifically for this project. We have added a sentence to the Methods (p.17) to make this explicit, citing Zugman et al. (2022):

> "*We meta-analysed data from 14 international cohorts (N = 693,869); 13 PGC-ANX studies with generalised anxiety symptom data, and summary statistics from a pre-existing GWAS[26]. We performed a meta-analysis with access to individual participant data[59], such that each PGC-ANX cohort performed genome-wide association analyses specifically for this study and shared summary statistics with the core analytical team.*"
